# Repurposing lurasidone to alleviate doxorubicin-induced cardiotoxicity and neurotoxicity via BDNF/TrkB/PI3K/Akt/CREB and miR-34a-5p/PGC-1α pathways

**DOI:** 10.1007/s00210-026-05019-z

**Published:** 2026-03-31

**Authors:** Nermeen A. Bayoumy, Abeer Elkhoely, Shimaa K. Mohamed

**Affiliations:** 1 National Cancer Institution, Cairo University, Kasr El-Aini, Cairo, 11562 Egypt; 2 Department of Pharmacology and Toxicology, Faculty of Pharmacy, Helwan University, Ein Helwan, Cairo, 11795 Egypt

**Keywords:** Doxorubicin, Lurasidone, Neurotoxicity, Cardiotoxicity, Oxidative stress, Inflammation, BDNF, MiR-34a-5p, PCG-1 alpha

## Abstract

Doxorubicin (Dox) is a potent cytotoxic medication, yet its adverse properties are undeniable obstacles to its clinical use. The objective of the existing research was to inspect the potential beneficial actions of lurasidone (Lura) against the neurotoxicity and cardiotoxicity triggered by Dox in rats. Sixty rats were equally allocated to four groups: Control group; Dox group; Lur (1 mg/kg) + Dox group; Lura (3 mg/kg) + Dox group. For 18 days, Lura (1 and 3 mg/kg) was given orally, starting 7 days before giving six doses of Dox (2.5 mg/kg every other day, i.p). Lura attenuated Dox-instigated cardiac injury as assured by the decrease in cardiac troponin-I (cTn-I), kg) and creatine kinase MB (CK-MB) levels. In addition, Lura remarkably declined Dox-triggered neuronal dysfunction, as confirmed by diminished anxiety and depression-alike behaviors in the open field (OFT) and forced swimming (FST) tests, respectively. Furthermore, Lura replenished cardiac and brain antioxidant markers, mitochondrial modulator, PGC-1α, and significantly decreased inflammatory mediators, miR34a-5p, and pro-apoptotic caspase-3 levels. In the brain, Lura also mitigated the induction of glial fibrillary acidic protein (GFAP) and ionized calcium binding adaptor-1 (Iba-1). In the same context, Lura pretreatment upregulated the brain-derived neurotrophic factor (BDNF)/tropomyosin receptor kinase B (TrkB)/phosphoinositide 3-kinase (PI_3_K) alleyway, along with the downstream proteins, the phosphorylated form of kinase B (p-Akt), and phosphorylated cAMP-response element binding form (p-CREB). The previously mentioned results were confirmed by histological examination and toluidine blue staining. Taken together, Lura conferred its cardioprotective and neuroprotective effects on Dox-treated rats through the enhancement of the BDNF/TrkB/PI3K/Akt/CREB signaling pathway, along with downregulation of miR 34a-5p leading to alleviated oxidative stress, inflammation, mitochondrial dysfunction, and apoptosis.

**Supplementary Information:**

The online version contains supplementary material available at 10.1007/s00210-026-05019-z.

## Introduction

Doxorubicin (Dox), an anthracycline drug, is perceived as an influential cytotoxic agent against several malignancies. Nevertheless, the therapeutic manipulation of Dox is precluded by its catastrophic, almost inevitable downsides, namely, cardiotoxicity, neuropathy, renal injury, and myelosuppression (Jordon 2002). The cascades of Dox-drawn cardiotoxicity are proposed due to oxidative stress enhancement, decreased synthesis of nucleic acids and proteins, release of vasoactive amines, and dysregulated adrenergic function (Minotti et al. 2004).

Moreover, Dox provokes neurological deficits, which are characterized by cognitive impairment (Du et al. 2021; Ongnok et al. 2021; Wang et al. 2021). On the same context, Dox does not penetrate the blood–brain barrier (BBB); instead, peripherally, Dox results in the elaboration of oxidative stress-related constituents, inflammatory mediators, and neurotransmitters, which collectively compromise the BBB integrity, thus entering the CNS leads to microglial and astrocyte activation. Following activation, microglial and astrocyte cells release large amounts of inflammatory cytokines and ROS and enhance GFAP and Iba-1 expression, which eventually result in neuronal death and a decline in neurogenesis (Tangpong et al. 2006; Aluise et al. 2011; Liu et al. 2017; El-Agamy et al. 2018; Allen et al. 2019).

One of the most prevalent neurotrophins in various mammalian tissues is brain-derived neurotrophic factor (BDNF) (Chao et al. 2006). Generally, it is identified as playing a pivotal part in neuronal cell viability and in preventing neuronal deterioration (Li et al. 2022).

Interestingly, BDNF is likewise conveyed in endothelial cells and smooth muscle, namely cardiac tissue. A preceding work has publicized that BDNF cascade is indispensable for proper murine cardiac contraction and relaxation (Feng et al. 2015).

Moreover, BDNF has been shown to confer neuroprotection and cardioprotection by binding to its specific receptor (TrkB) and subsequently activating protein kinase B (Akt) signaling (Hang et al. 2017, 2021; Liao et al. 2020), ultimately engaging phosphoinositide 3-kinase (PI3K)/protein kinase B (Akt) signaling, which plays a decisive role in regulating neuroprotection and cardioprotection by upregulating anti-apoptotic proteins and combating oxidative stress (Sakamoto et al. 2011; Xu et al. 2016).

Additionally, preceding research has displayed a correlation between specific miRNA levels and some disease processes, such as stroke, cardiac hypertrophy, and myocardial infarction (Zhang et al. 2022; Payne et al. 2023). Among the furthermost essential miRNAs is miR-34a due to its role in modulating mitochondrial functions (Payne et al. 2023). Numerous studies have indicated the magnitude of the role miRNAs play in maintaining the integrity and functionality of mitochondria. A member of the miR-34 family, miR-34a-5p is a well-researched p53-dependent tumor suppressor that takes part in ATP synthesis and mitochondrial oxidative phosphorylation (Tai et al. 2021). Likewise, there is mounting evidence that the pathological mechanistic pathways underlying age-related morbidities are associated with elevated levels of miR-34a-5p, which are typically allied with the dysfunction and decreased autophagy (Catanesi et al. 2020; Tai et al. 2021). Moreover, previous work showed the significant role of miR-34a-5p in mitophagy through modulating sirtuin-1 (SIRT1)/*mammalian target of rapamycin (*mTOR) signaling pathway, which affects the expression of *PTEN-induced kinase 1* (PINK1) and peroxisome proliferator-activated receptor gamma coactivator 1-alpha (PGC-1α) (Thounaojam et al. 2019; Tai et al. 2021).

Lurasidone (Lura) is an antipsychotic medication used to manage various mental illnesses, specifically schizophrenia and bipolar depression (Ishibashi et al. 2010). Previous studies have revealed Lura’s antioxidant effects (Biswal et al. 2015; Spero et al. 2022). Moreover, it was documented that Lura boosts BDNF expression (Fumagalli et al. 2012).

The objective of the existing study was to explore the feasible shielding efficacy of Lura against Dox-promoted cardiotoxicity and neurotoxicity in rats. In the same context, the study was extended to explicate the impression of Lura on BDNF expression and its downstream signaling pathways and the implication of these pathways in conferring cardio- and neuroprotection.

## Materials and measures

### Animals

The National Cancer Institute’s animal house in Cairo, Egypt, housed 60 male Sprague Dawley rats weighing in the range of 200–250 g. Temperature-controlled surroundings (25 °C ± 2 °C) were used for rat housing. For 1 week prior to the investigational measures, rats were offered unrestricted access to the pelleted nourishment and purified drinking water. Animal management robustly adhered to the Use and Care of Laboratory Animals legislation, which was enacted by the Ethics Research Board of the Faculty of Pharmacy, Helwan University, and conforms to ARRIVE guidelines. Every attempt has been made to lessen each animal’s suffering by following an authorized experimental protocol (IACUC No. 02A2022).

### Cell line

The National Cancer Institute in Cairo, Egypt, is the source of the human breast cancer cell line (MCF-7), which was originally obtained from the American Type Culture Collection (ATCC, Manassas, VA, USA). A total of 1.5 g/L sodium bicarbonate, 10% FBS, 2 mmol/L l-glutamine, 100 g/L streptomycin, and 100 U/mL penicillin were added to the RPMI-1640 medium in which the cells. At 37 °C, the cells were cultivated in an atmosphere of 95% air and 5% CO2.

### Drugs and chemicals

Doxorubicin (DOX) was obtained from Mylan (Rockford, USA). Lurasidone (Lura) was donated as a gift from AL-Andalus Pharmaceutical Company (Egypt).

### Network analysis

To ensure robustness and reproducibility, the network pharmacology approach was implemented using a well-established pipeline, as detailed earlier by Mohsen et al. (2025) and El Tabaa et al. (2025).

#### Network-based target identification for lurasidone and doxorubicin

To initiate the network pharmacology workflow, the chemical structures of lurasidone and doxorubicin were acquired in SMILES format from the PubChem database (Supplementary Table S1, https://pubchem.ncbi.nlm.nih.gov/) (Kim et al. 2025).

Subsequently, a comprehensive search for experimentally validated and predicted protein targets was conducted across these databases as follows: (1) Comparative Toxicogenomics Database (CTD, http://ctdbase.org/) (Davis et al. 2023); (2) The SwissTargetPrediction database (http://www.swisstargetprediction.ch/) (Daina et al. 2019); and (3) Open target platform (https://platform.opentargets.org/) (Ochoa et al. 2023), with all queries performed on 6 September 2025. To ensure data uniformity for downstream analyses, all retrieved targets were mapped to their official gene symbols based on the UniProtKB (https://www.uniprot.org/) (UniProt: the Universal Protein Knowledgebase in 2023, 2023), and any redundant entries were eliminated.

#### Selection of cardiotoxicity- and neurotoxicity-linked gene targets

A systematic search was conducted to identify potential targets related to cardiotoxicity and neurotoxicity using the following public databases: (1) Comparative Toxicogenomics Database (CTD, http://ctdbase.org/, accessed on 6 September 2025).

Broad toxicity-related gene lists were initially retrieved from the CTD to ensure comprehensive coverage of all reported cardiotoxicity and neurotoxicity associations. These large datasets were intentionally used as an unbiased starting point, with subsequent intersections with Dox and Lura targets and the final experimental validation steps serving as confidence-filtering processes to refine the gene set to high-confidence targets.

; (2) GeneCards (https://www.genecards.org, accessed on 6 September 2025) (Stelzer et al. 2016); (3) Open Targets Platform (OTP, https://platform-docs.opentargets.org/, accessed on 6 September 2025) (Ochoa et al. 2021). Queries were performed on 6 September 2025 using the keywords “cardiotoxicity” and “neurotoxicity.” All retrieved targets were mapped to their official gene symbols according to the UniProt knowledgebase, and any redundant entries were eliminated.

#### Definition of doxorubicin toxicity targets

Targets associated with doxorubicin-induced toxicity (DIT) were compiled by merging the sets of targets implicated in doxorubicin-induced cardiotoxicity (DIC) and neurotoxicity (DIN). The DIC and DIN target sets were each defined by detecting the intersection between the curated doxorubicin protein targets and the independently curated sets of targets associated with cardiotoxicity or neurotoxicity, respectively.

#### Construction of the protein–protein interaction (PPI) network

The similar targets of lurasidone and DIT were first analyzed using a Venn diagram, constructed with the jvenn online implement (https://jvenn.toulouse.inrae.fr/app/index.html) (Bardou et al. 2014). Subsequently, these common targets were submitted to the STRING database Version 12.0 (https://string-db.org/, accessed on 6 September 2025) (Szklarczyk et al. 2023) to generate a PPI network. The network parameters were determined at a medium confidence score (0.4). The network was expanded by specifying a maximum of 50 interactors for the first shell and a further 50 interactors for the second shell. Disconnected nodes were omitted from the final network.

Following PPI construction, a comprehensive drug-disease-target-pathway network was built. All networks were visualized with Cytoscape software 3.10.2 (NIGMS, United States) (Shannon et al. 2003), and nodes were ranked by their degree of centrality, as calculated by the CytoNCA plugin (Tang et al. 2015).

#### Gene ontology and pathway enrichment analysis

Enrichment analysis for Gene Ontology (GO) terms (biological process, molecular function, cellular component) and Kyoto Encyclopedia of Genes and Genomes (KEGG) pathways was conducted with ShinyGO (http://bioinformatics.sdstate.edu/go/#tab-6415-3, accessed on 6 September 2025) (Ge et al. 2020). Through a false discovery rate (FDR) threshold of < 0.05, the Significant terms were recognized. The results, visualized as horizontal lollipop plots, are provided by the ShinyGO tool.

### Experimental setup

Four groups of 60 rats were sporadically and equally assigned. The doses of Dox (2.5 mg/kg, i.p.) and Lura (1 and 3 mg/kg, p.o.) were picked rendering on earlier studies (Enomoto et al. 2008; Ramalingayya et al. 2017; Siswanto et al. 2016; Liao et al. 2022). Animals were treated for 18 days according to the regiment: *Group I:* Control group was given a daily dose of an i.p. injection of normal saline. *Group II:* Dox group administered six consecutive doses of Dox (2.5 mg/kg, i.p.), diurnal upon diurnal, starting from the 8th day of the experiment. *Group III* Lura 1 mg + Dox group and *Group IV* Lura 3 mg + Dox, groups were given a daily dose of Lura (1or 3 mg/kg, p.o.) for 18 days, starting 7 days before the administration of 6 consecutive doses of Dox (2.5 mg/kg, i.p.), diurnal upon diurnal. On the 19th day of the investigation, the habituation of rats for the behavioral tests was performed, and on the 20th day, a behavioral assessment was carried out. Blood specimens were then drawn through the retro-orbital zone after anesthesia with ketamine (100 mg/kg/i.p) (Mosaad et al. 2025). The serum was collected subsequently centrifuging the blood for about 15 min at 3000 rpm and kept stored in − 80 °C to be used for assessment. Afterward, rats were forfeited.

**Fig. 1:**
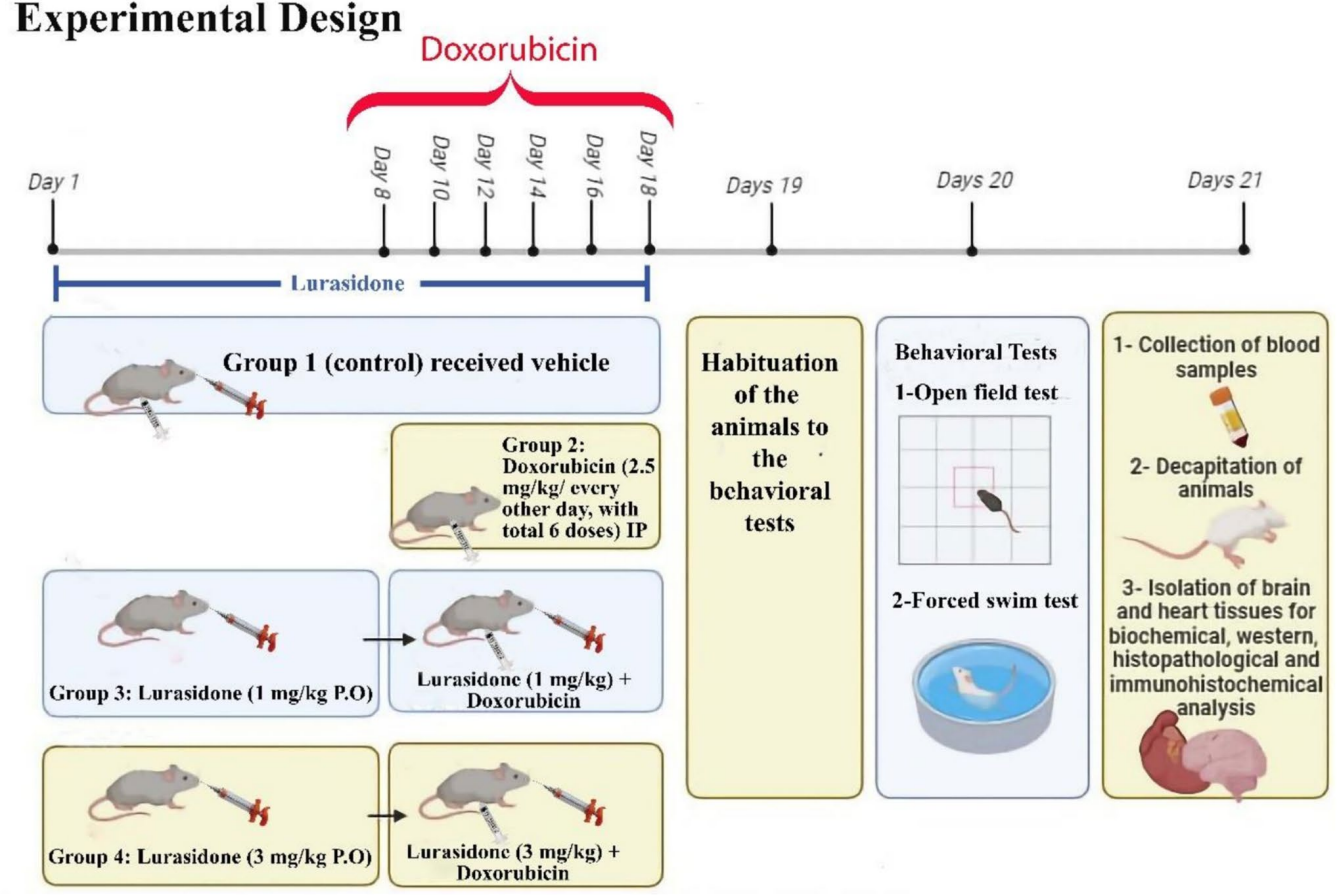
The experimental design of the work

The brain and heart samples were instantaneously taken off and rinsed in an ice-cold saline liquid. Three specimens were entrenched in paraffin blocks from each group and conserved in 10% formalin for histological analysis and immunochemistry investigation. Six brains and hearts from all sets were smoothed in phosphate buffer saline, and the clear supernatants were utilized for biochemical analysis. Finally, the last six brains and hearts were used for western blot investigation from each set (Fig. 1).

### Assessment of behavior

#### Open field test (OFT)

The OFT was held in an 80 × 80 × 40 cm square wooden container with red interior stockades and a black smooth polished base. All groups were subjected to open-field tests on the 20th day of the experiment. Each rat was carefully placed in the center of the wooden box, and then its behavioral features were assessed for 3 min. The following behavioral parameters were recorded during the set duration: rearing (the number of times the animal raised on its forelimbs), ambulation frequency (the number of squares traversed by the animal), and grooming frequency (Sun et al. 2019).

#### Forced swimming examination

The forced swim test (FST) is a well-known archetype for the evaluation of antidepressants. Rats are compelled to swim in a cylinder with no exit, and their behavior is evaluated as active (swimming and climbing) or inactive (immobility). The height of water is set at 15 cm from the base of the cylinder so that the water is deep enough that the animal’s limbs and tail do not rest on the bottom. The animal strives to flee during the initial time of intensive activity. Eventually, the animal ceases to move aggressively and develops a distinctive immobility, moving to keep its head above water. This physical immovability is regarded as an indication of behavioral despair. On the 20th day of the experiment, all groups underwent the FST. Each rat was forced to swim for 5 min. The following behavioral variables were recorded during this time: immobility time and swimming time (Yankelevitch-Yahav et al. 2015). An investigator blind to the treatment conditions conducted all the behavioral trials and data collection.

### Biochemical measurements

#### Quantitative determination of serum creatine kinase MB (CK-MB) and cardiac troponin-I (cTn-I) levels

CUSABIO Enzyme-linked Immunosorbent Assay Kit (Cat. No CSB-E08594r, USA) and Cloud Clone Corpe (Cat. No. SEA479Ra, USA) were utilized for the revealing of cTn-I, and CK-MB levels in serum, respectively.

#### Quantitative determination of dopamine (DA) and acetylcholinesterase levels (AchE) in brain tissue

MyBioSource ELISA kits (USA) were used to detect DA and AchE levels in the brain tissue (Cat. No. MBS725908 and MBS2709297, respectively). The guidelines provided by the manufacturer were followed for each procedure.

#### Reduced glutathione (GSH) content and superoxide dismutase (SOD) activity determination in heart and brain tissues

Bio diagnostic and research reagents (CAT. No. SD 25 21, GR 25 11) were used to assess GSH content and SOD activity in the heart and brain tissues using the colorimetric method.

#### Estimation of nuclear factor-kappa B (NF-κB), peroxisome proliferator-activated receptor gamma coactivator 1-alpha isoform 1 (PCG-1α), tumour necrosis factor (TNF-α), and interleukin 1 (IL-1β) contents of heart and brain tissues

MyBioSource ELISA kit (Cat. No. MBS453975, MBS3808684, USA) and Cloud Clone Crop (Cat. No. SEA133Ra, SEA563Ra, USA) were employed to detect NF-κB, PCG-1α, TNF-α, and IL-1β contents in the heart and brain tissues. All procedures were performed as instructed by the vendor.

#### Determination of the anti-apoptotic marker caspase 3 (CASP3) level in heart and brain tissues

An Enzyme-linked Immunosorbent Assay Kit, Cloud Clone Crop (Cat. No. SEA626Ra, USA), was used to determine caspase-3 levels in heart and brain samples. Assessments were implemented in accordance with the vendor’s guidelines.

#### Assessment of the p50 subunit of NF-ĸB, BDNF, TrkB, p-AKT, and p-CREB using Western blotting in heart and brain tissues

Following lysis with the ReadyPrepTM protein extraction kit (entire protein) obtained from Bio-Rad Inc. (Catalogue #163–2086), the Bradford Protein Assay Kit (SK3041) from Bio Basic Inc. (Markham, Ontario L3R 8T4 Canada) was used to quantify the extracted proteins from the tissues. Protein denaturation was achieved by polyacrylamide gel electrophoresis utilizing the TGX Stain-FreeTM FastCastTM Acrylamide Kit (SDS-PAGE), a product of Bio-Rad Laboratories Inc. with Cat. # 161–0181. Afterwards, the BioRad Trans-Blot Turbo was used to relocate protein bands from the gel to the membrane. The membrane was subsequently clogged for an hour at room temperature with tris-buffered saline containing 3% bovine serum albumin (BSA) and Tween 20 (TBST) buffer. According to the producer’s instructions (catalogue numbers: sc-8414, sc-293125, sc-81486, sc-377218, and sc-65514, respectively), primary antibodies for p50 NF-ĸB, p-AKT, p-CREB, TrKB, and BDNF were diluted in TBST.

Subsequently, membranes were incubated with an HRP-conjugated secondary goat anti-rabbit antibody. The chemiluminescent substrate Clarity TM Western ECL from Bio-Rad (cat#170–5060) was added to the blot as instructed by the included commands.

An image based on a CCD camera was applied to see the protein bands, then normalized by utilizing β-actin protein expression as an internal standard, and the images were examined with a Chemi Doc MP imager.

### Estimation of Mir34a-5p by quantitative reverse transcription polymerase chain reaction (QRT-PCR)

SV Total RNA Isolation System (Promega, Madison, WI, USA) was exploited to obtain total RNA from a tissue homogenate. The cDNA was obtained by transforming whole RNA (0.5–2 µg) via an extreme-capacity cDNA reverse transcription kit (Cat. No. K1621, Fermentas, USA). Version 3.1 of the Applied Biosystem software (StepOne™, USA) was utilized for real-time qPCR amplification and investigation. The annealing temperature was optimized for the qPCR procedure using the primer sets. The primers employed were as illustrated: AT-1R: forward: 5′-ACACTCCAGCTGGGTGGCAGTGTCTTAGCT-3′, reverse: 5′-TGGTGTGGAGTCG-3′; β-actin: forward: 5′-GAGACCTTCAACACCCCAGC-3′, reverse: 5′-ATGTCACGCACGATTTCCC-3′.

### Immunohistochemical analysis

Biological examination was achieved as per Abbas et al. (2021); Leica application system modules (Leica, Microsystems GmbH, Wetzlar, Germany) were employed for histological assessment. The relative mean positive area percentage of GFAP, induction of caspase-3, and the number of positive Iba-1 reactive microglia (Cat#13–0300, 700,182, and MA5-29,012, respectively; Thermo Fischer Scientific Co.) were assessed. Moreover, the expression of p-PI3K was determined (Cat#bs-6417R-Bioss, USA) by scanning, segmenting, and analyzing six randomly picked, non-overlapping fields on each slide (Abbas et al. 2021).

#### Histopathological evaluation

Rat brain and heart tissue samples were irrigated then, anchored for 48 h with 10% neutral buffered formalin before placing them in paraffin wax. Following exposure to ascending concentrations of ethanol, the brain and heart samples were cleaned in xylene, permeated, and inserted in Leica Biosystems’ Paraplast plus tissue-embedding solution. After that, tapping a rotatory microtome, thickness of 4-μm serial sagittal brain slices were cut and mounted on glass slides to demonstrate the hippocampus (CA3) in diverse samples. Heart tissue samples, as well, were carved into 5 µm slices by a rotary microtome. Hematoxylin and eosin were used to dye the slices for histological investigation underneath a light microscope (Culling 1974). In addition, Toluidine blue staining is used to determine the number of intact neuron cells. A light microscope (Leica Microsystems GmbH, Wetzlar, Germany) was used to snap the images, and Adobe Photoshop version 8.0 was used for inspection. Applying the Leica Qwin 500 Image Analyzer (LEICA Imaging Systems Ltd., Cambridge, England), morphometric assessment was carried out on the stained slides. At a 400 × magnification power, the count of intact neuron cells was considered in six disciplines per slide.

#### Cytotoxicity assay

The Sulforhodamine B (SRB) test was used to assess the IC_50_ (half-maximal inhibitory concentration) of Dox alone and in the presence of a subtoxic concentration (IC_10_) of Lura (Skehan et al. 1990). Initially, 200 µl of fresh medium was used to seed cancer cells at a concentration of 4 × 10^3^ cells/well in 96-well microtiter plates, and the cells were allowed to adhere to the plates. The media were changed after 24 h and supplemented with fresh medium containing the appropriate quantities of the drug. Dox was used at 5–50 µg/ml, while Lura was used at the subtoxic concentration (IC_10_) of 5 µg/ml for the MCF-7 cell line. After a whole day of treatment, the cells were fixed for 1 h at 4 °C with 10% trichloroacetic acid. The plates were washed with distilled water using an automatic washer (Tecan, Germany) and stained with 50 µl of 0.2% SRB dissolved in 1% acetic acid for about 30 min at room heat and in the dark.

The colorant was then melted by 200 µl/well of 10 M tris base (pH 10.5), and spectrophotometric measurement of the optical density of every well was achieved via an ELISA microplate reader (Sunrise Tecan reader, Germany) at 570 nm. Using Graph Pad, Prism software, version 9; GraphPad, San Diego, CA, USA, concentration–response blots were obtained, and the IC50 for each curve was computed.

#### Statistical analysis

All records were indicated as means ± standard deviations (SD). For entire parameters, one-way ANOVA was employed, except for the survival fraction of MCF-7, where two-way ANOVA accompanied by Tukey’s multiple comparison analysis was used. GraphPad Prism (version 9) was used to execute all statistical analyses. The significance threshold was established at *p* < 0.05 for all arithmetical examinations.

## Results

### Network analysis

#### Identification of lurasidone’s molecular targets

Initial screening yielded 109 potential lurasidone targets: 7 from the CTD, 100 from the SwissTargetPrediction database, and 2 from the open target platform. After data integration and the removal of duplicates, 101 unique targets were retained for further analysis (Supplementary Table S2).

#### Identification of mechanisms in DIT

Total of 9101 putative protein targets of doxorubicin were compiled from three primary sources: CTD: 9000, the SwissTargetPrediction database: 100, and the Open Targets Platform: 1. Following data integration and the removal of duplicates, a non-redundant set of 9039 unique doxorubicin targets was established for subsequent analysis.

Targets associated with cardiotoxicity and neurotoxicity were similarly aggregated. The cardiotoxicity target set was constructed from CTD: 34,527, GeneCards: 485, and OTP 31. After deduplication, 34,566 unique targets associated with cardiotoxicity were retained.

The neurotoxicity target set was sourced from CTD: 48,617, GeneCards: 2969, and OTP: 405, resulting in a final deduplicated set of 48,721 targets (Supplementary Table S3).

To elucidate the mechanisms underlying doxorubicin’s adverse effects, the molecular targets for DIC were defined as the intersection between the curated doxorubicin target set and the cardiotoxicity-associated target set (Supplementary Table S4). An identical analytical procedure was applied to identify the targets implicated in DIN (Supplementary Table S4). Deduplicated targets associated with DIT were then compiled by merging the resulting DIC and DIN target sets. This integrated approach yielded a unified set of high-confidence targets putatively responsible for the drug’s adverse effects.

**Fig. 2:**
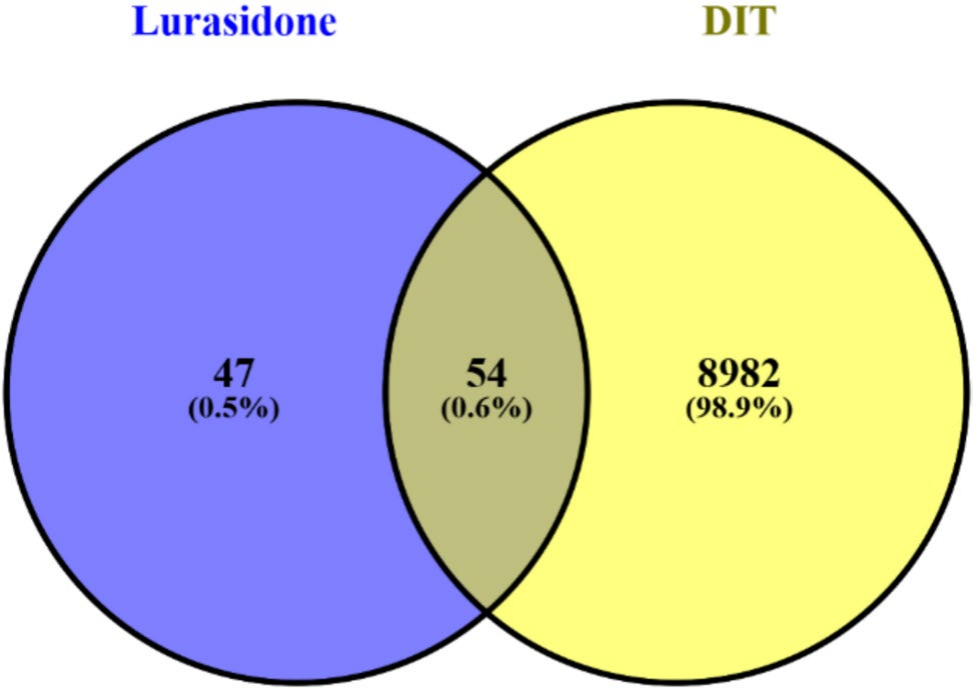
Venn diagram of the intersecting targets for lurasidone and doxorubicin-induced toxicity (DIT)

The molecular targets of lurasidone were cross-referenced with the target sets DIT. This analysis identified 54 shared targets between lurasidone and DIC, and 54 shared targets between lurasidone and DIN (Fig. 2), as detailed in Supplementary Table S5.

#### Construction and analysis of the PPI network

**Fig. 3:**
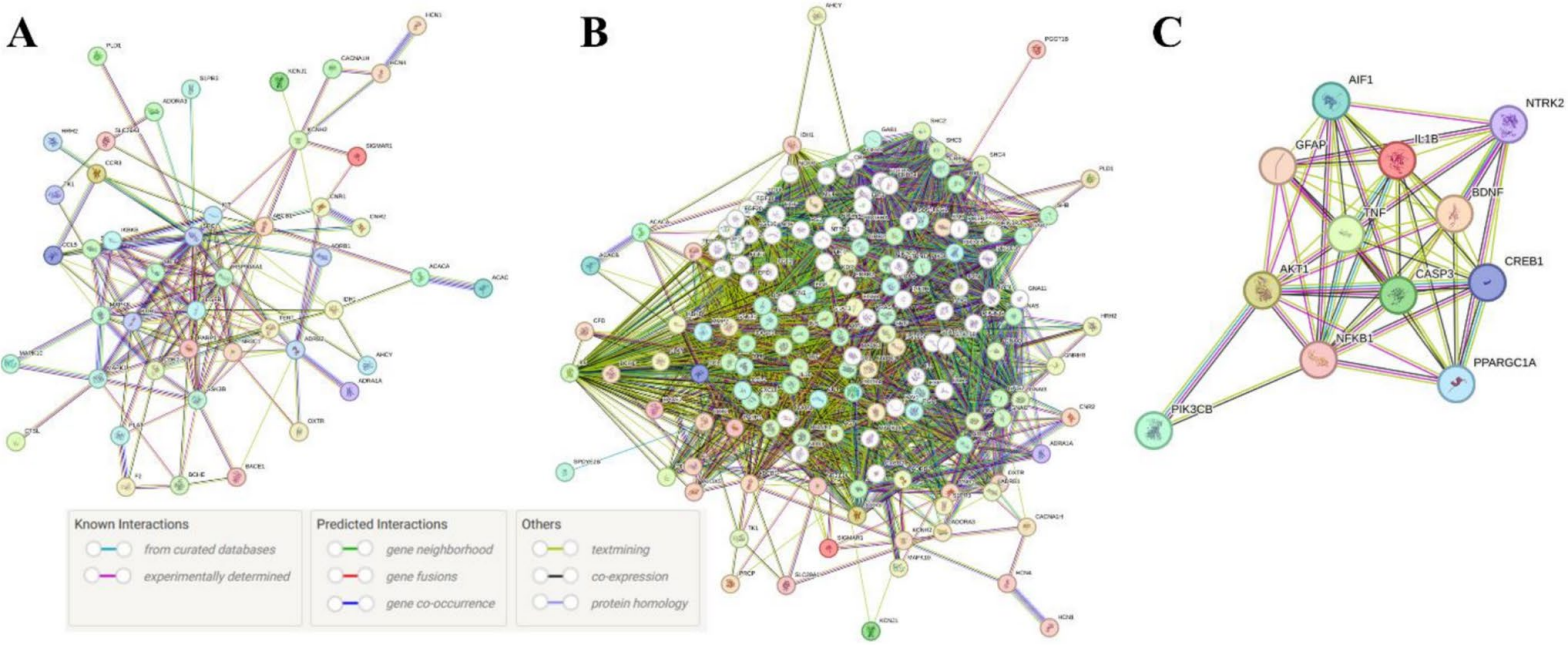
Network analysis of common targets, as generated by the STRING database. A The core PPI network of the 54 common target proteins, comprising 54 nodes and 150 edges. B The expanded PPI network after the integration of 100 top first- and second-shell interactors, resulting in a comprehensive network of 154 nodes and 4309 edges. C PPI network of the twelve experimentally validated targets generated

To investigate interactions among the 54 common targets, a PPI network was generated using the STRING database. The core network contained all 54 query targets as nodes, connected by 150 edges (Fig. 3A). To capture a more comprehensive and biologically relevant interactome, the network was expanded by 100 first- and second-shell interactors. This yielded a significantly denser network of 154 nodes and 4309 edges (Fig. 3B), which was used for subsequent analysis.

Given that the six pathway-related targets were implicated by enrichment analysis, we hypothesized that they functionally interact with the core target set. To test this hypothesis and elucidate the functional relationships and potential crosstalk, a PPI network was constructed among all twelve targets. Interaction edges among the selected proteins indicate extensive crosstalk between inflammatory mediators and neurotrophic pathways (Fig. 3C).

Table 1 lists the six hub targets with their corresponding degree values, identified in the context of doxorubicin-induced cardiotoxicity and neurotoxicity. These targets were selected due to their high connectivity, indicating pivotal roles in mediating key pathological processes triggered by doxorubicin. These hubs are critically involved in the regulation of oxidative stress, inflammation, apoptosis, and neuroprotection pathways affected during doxorubicin toxicity. Node importance was assessed by ranking the nodes based on their degree centrality, calculated using the CytoNCA plug-in (Supplementary Table S6).

**Table 1:** Identification of hub nodes based on degree centrality

Rank	Target name	Degree
2	**AKT1**	**117**
8	**TNF**	**102**
17	**CASP3**	**91**
19	**IL1B**	**91**
34	**BDNF**	**84**
64	**PIK3CB**	**71**

#### Network pharmacology analysis reveals interactions between drug, disease, targets, and pathways

**Fig. 4:**
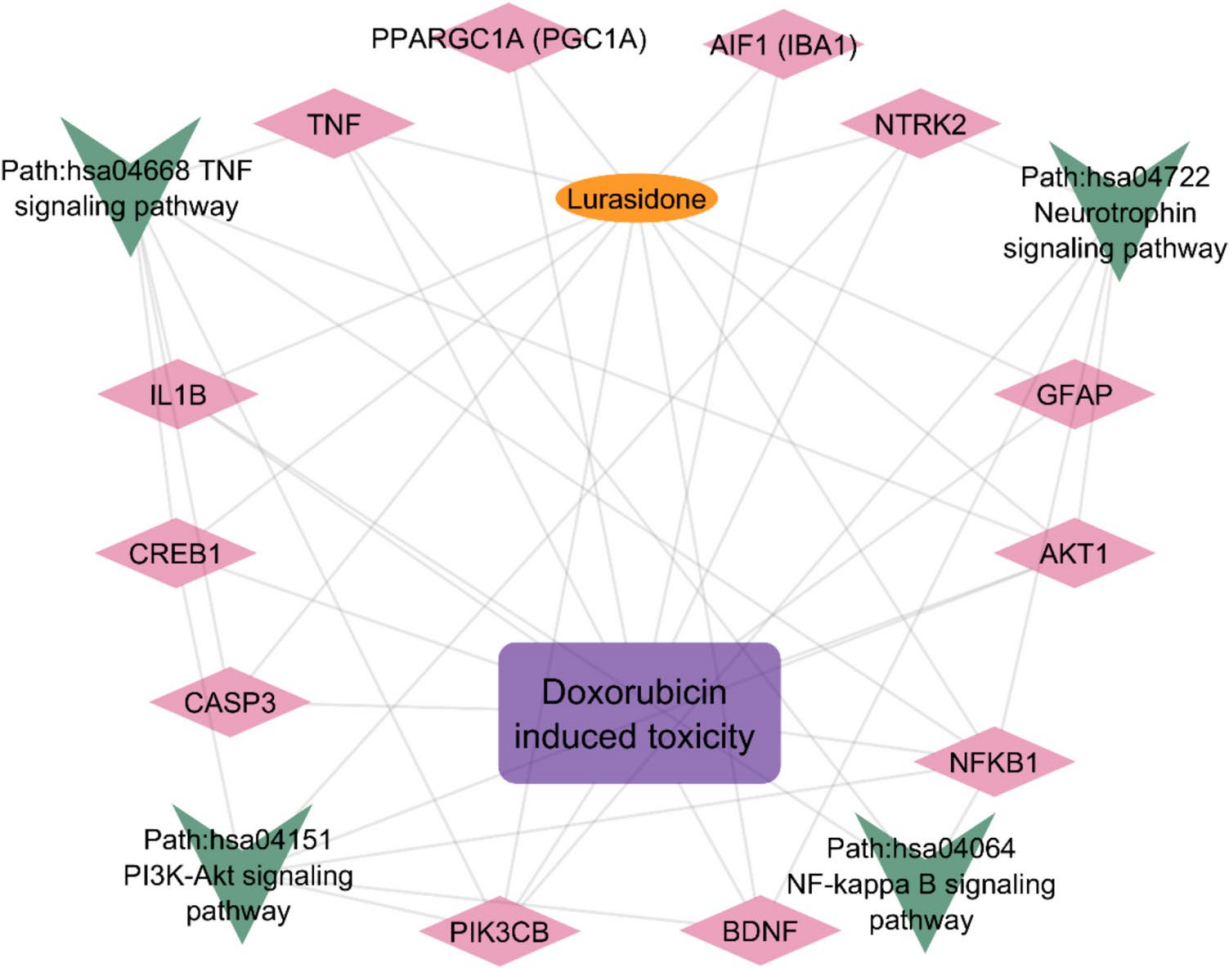
Integrated drug-disease-target-pathway network, ellipse: drug, rectangle: disease, diamond: targets, V-shape: pathways

To visualize the interactions, an integrated drug-disease-target-pathway network was generated in Cytoscape (Fig. 4).

#### Enrichment analysis reveals key signaling pathways

**Fig. 5:**
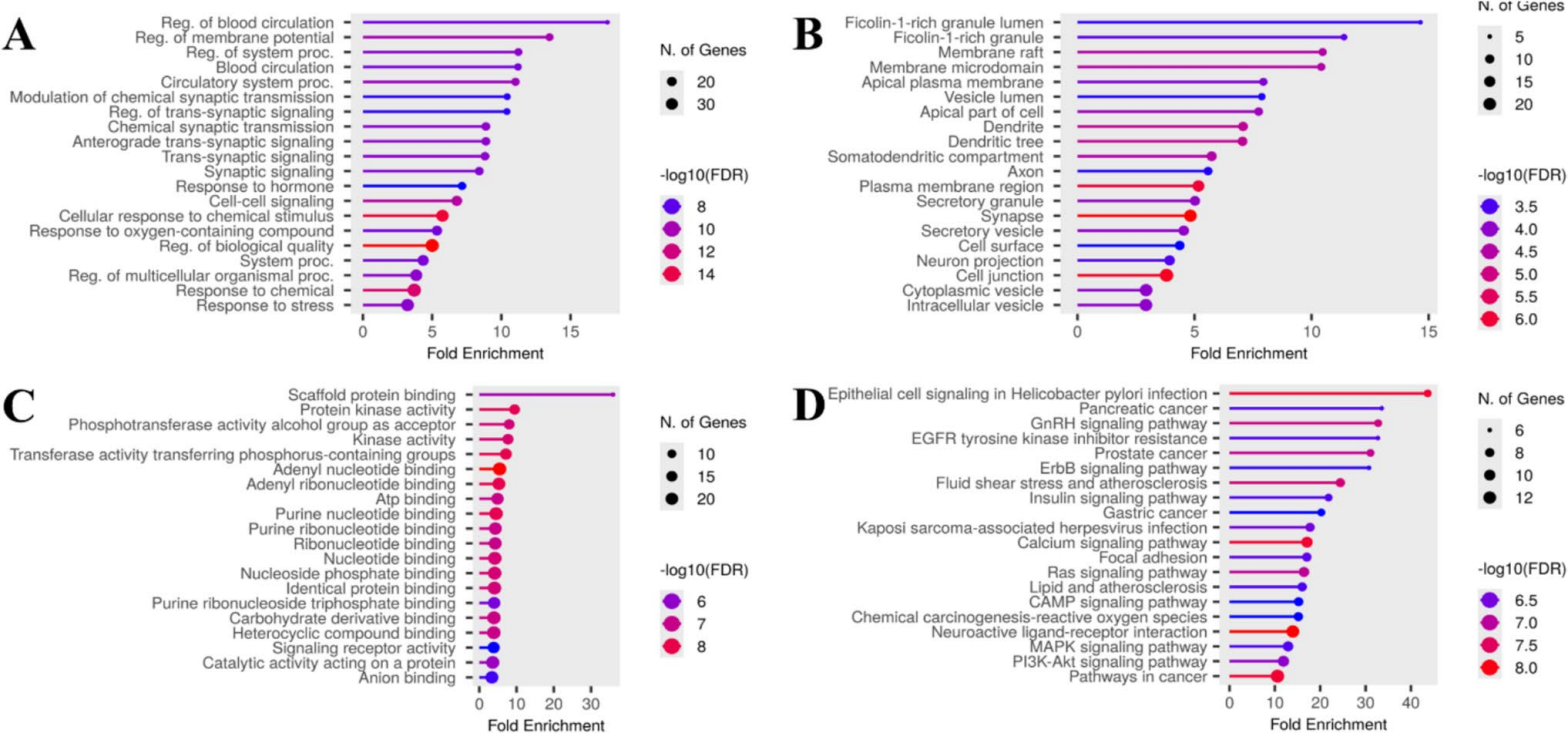
Top 20 significantly enriched analyses. **A **Biological process. **B **Cellular component. **C **Molecular function. **D **KEGG pathways. All are sorted by fold enrichment

Enrichment analysis positioned the 12 experimentally validated targets within biological pathways central to doxorubicin-induced toxicities. Functional enrichment analysis of the 154 candidate targets revealed significant associations with specific biological themes (Fig. 5). GO analysis (FDR < 0.05) identified top terms including regulation of biological quality, cellular response to chemical stimulus, and cell–cell signaling (biological process); synapse, cell junction, and dendrite (cellular component); and adenyl nucleotide binding and protein kinase activity (molecular function). KEGG pathway analysis identified 209 significantly enriched pathways.

Given the focus of this study on doxorubicin-induced toxicities, four key immune-inflammatory pathways were prioritized for further validation (Table 2). Consequently, the targets mapping to these pathways were selected for in vivo analysis.

**Table 2:** Key pathways derived from KEGG analysis

KEGG number	Pathway name	Fold enrichment
Path:hsa04722	Neurotrophin signaling pathway	42
Path:hsa04668	TNF signaling pathway	28
Path:hsa04151	PI3K-Akt signaling pathway	27
Path:hsa04064	NF-kappa B signaling pathway	18

A full listing of all significant GO terms and KEGG pathways is available in Supplementary Table S7.

#### Modulation of neurotrophin, TNF, PI3K-Akt, and NF-kappa B pathway targets

To elucidate the mechanism of lurasidone’s protection against doxorubicin-induced toxicity, we experimentally assessed the expression of key targets within the Neurotrophin, TNF, PI3K-Akt, and NF-kappa B signaling pathways. From the subset of common targets identified computationally between lurasidone and doxorubicin-induced toxicity, validation assays confirmed the significant modulation of six targets: IL1B, BDNF, AKT1, PIK3CB, TNF, and CASP3. Furthermore, analysis extended to six additional pathway-relevant targets not found in the common target pool revealed that AIF1 (IBA1), PPARGC1A (PGC1A), CREB1, NTRK2, NFKB1, and GFAP were also significantly altered following lurasidone treatment. This empirical validation confirms the direct involvement of these specific molecules and underscores the concurrent regulation of the four hypothesized pathways in the observed protective effect.

### Influence of Lura (1 and 3 mg/kg) on behavioral modulation in Dox-treated rats

**Fig. 6:**
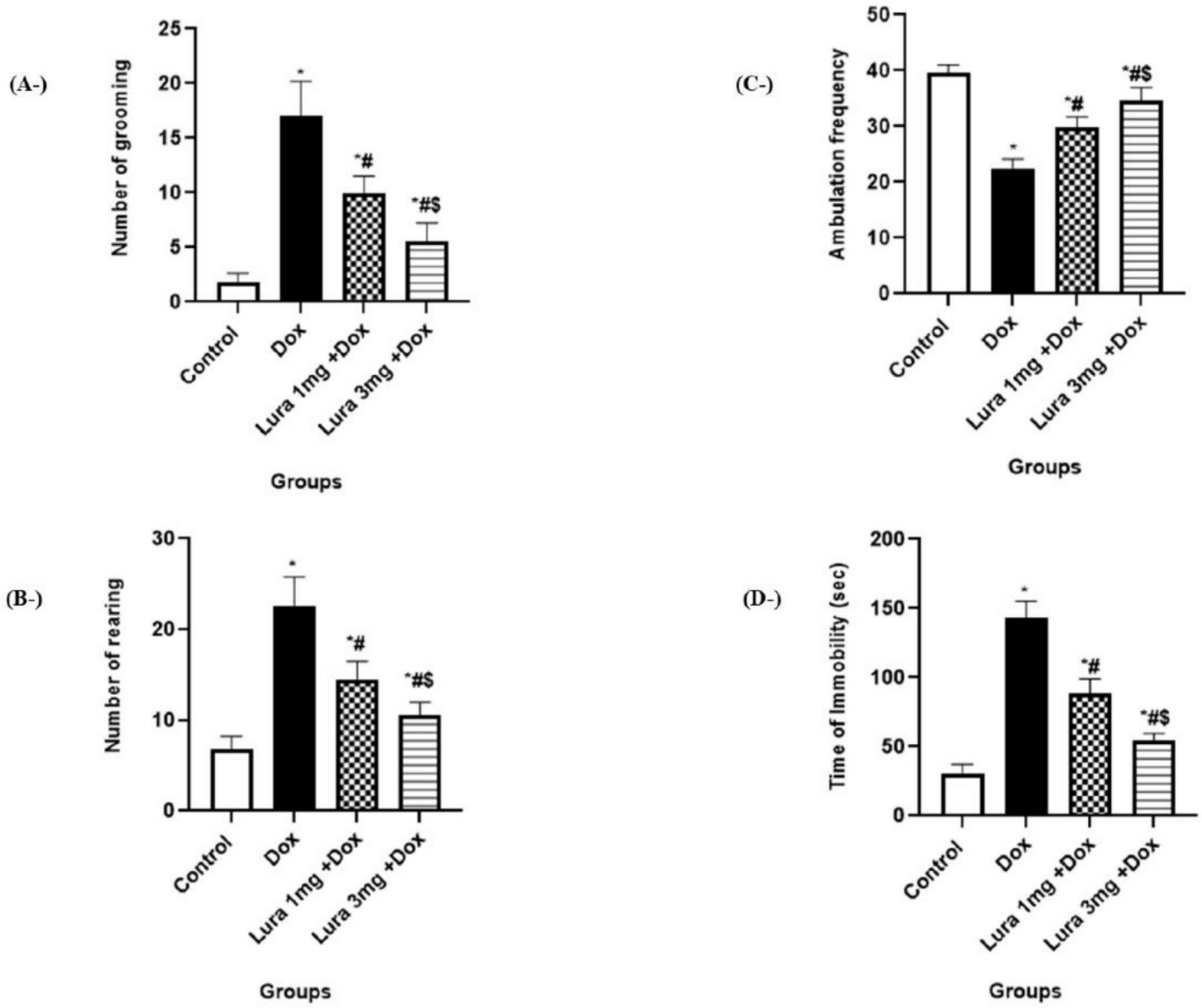
Effect of Lura (1 and 3 mg/kg) on behavioralchanges in Dox-treated rats.** A** Number of grooming in OFT. **B** Number of rearing in OFT. **C** Ambulation frequency in an OFT. **D** Time of immobility in FST. The outcomes have been reported by way of mean ± SD (*n* = 6). One-way ANOVA was used to test significance, ensued by Tukey–Kramer multiple comparisons. *: conveys statistical disparity from the control group; #: conveys statistical disparity from the Dox group; $: conveys statistical disparity from the Lura 1 mg + Dox group, at *p* < *0.05*. Dox: doxurubicin; Lura: lurasidone; FST: forced swimming test; OFT: open field test

Dox-induced anxiety-like behavior in rats was evaluated using the OFT. It has been demonstrated that Dox administration was accompanied by a remarkable (*p* < *0.05*) regression in locomotor activity by 43.35% and substantially boosted (*p* < *0.05*) the number of rearing and grooming by 2.3, 8.1-fold, respectively, when compared with normal rats. Lura (1 and 3 mg/kg) co-treatment established a notable enhancement (*p* < *0.05*) in locomotor activity by 32.93% and 53.98% and eventually a significant decline (*p* < *0.05*) in the number of rearing by 35.7% and 53.09%, and grooming by 41.57% and 67.05%, respectively, as relevant to the Dox-administered group (Fig. 6A, B, C).

Also, the Forced swimming test (FST) was performed to evaluate Dox-induced depressive-similar behavior in rats. As presented in Fig. 6D, Dox-treated rats exhibited a higher total time spent immobile (*p* < *0.05*) by 3.6-fold compared with normal rats. Contrastingly, pretreatment with Lura (1 and 3 mg/kg) notably down-regulated (*p* < *0.05*) the duration of immobility by 38.45% and 62.12%, respectively, compared with the Dox group.

### Consequence of Lura (1 and 3 mg/kg) on neuronal besides cardiac markers

**Fig. 7:**
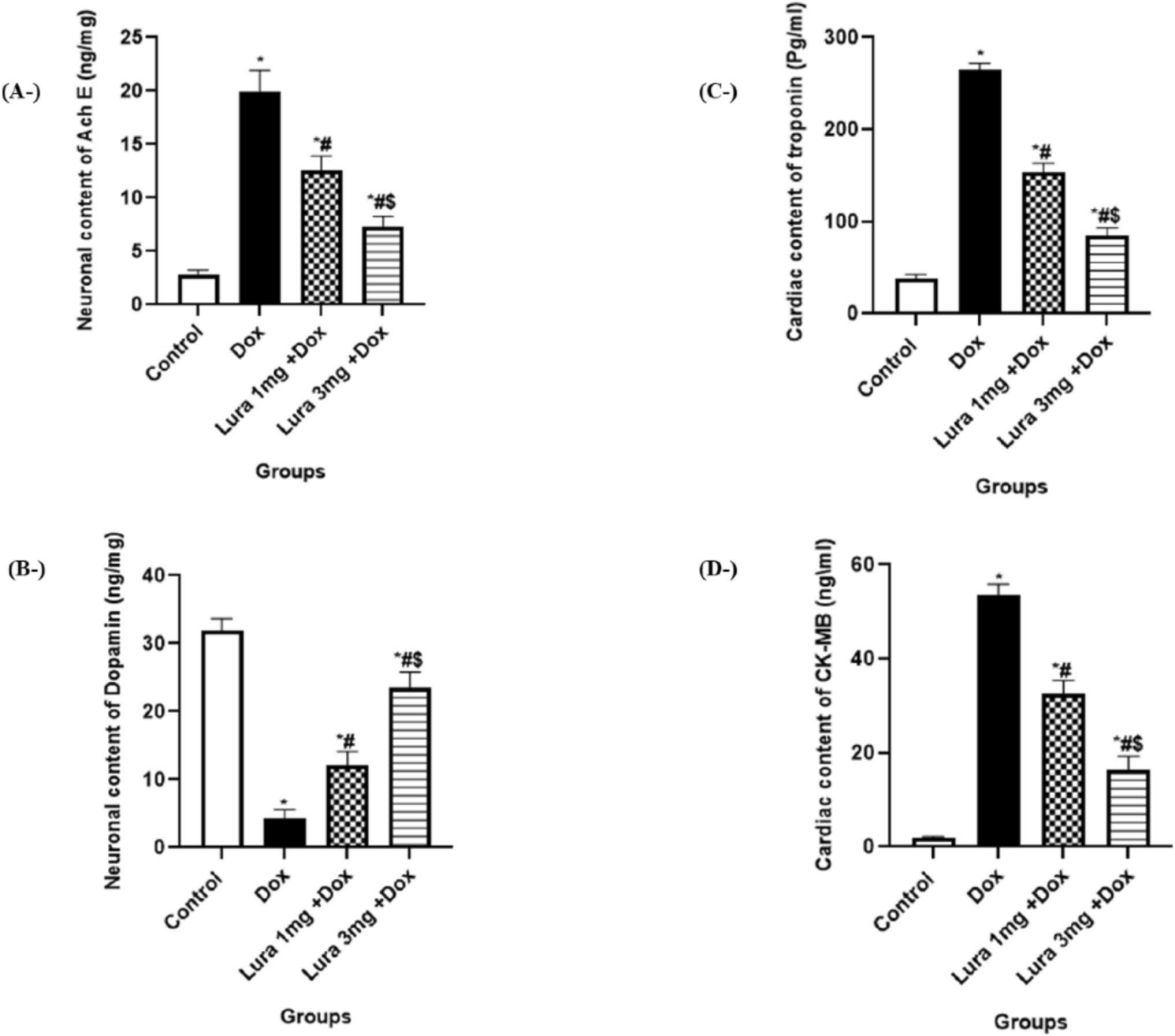
Effect of Lura (1 and 3 mg/kg) on neuronal and cardiac biomarkers in Dox-treated rats. Neuronal markers. **A** AchE. **B** DA; cardiac markers. **C** cTn-I. **D** CK-MB. The outcomes are reported as mean ± SD (*n* = 6). One-way ANOVA was used to test significance, followed by Tukey–Kramer multiple comparisons. *: conveys statistical disparity from the control group; #: conveys statistical disparity from the Dox group; $: conveys statistical disparity from the Lura 1 mg + Dox group at *p* < *0.05*. Dox: doxorubicin; Lura: lurasidone; AchE: acetylcholinesterase; DA: dopamine; cTn-I: cardiac troponin-I; CK-MB: creatinine kinase-MB

Our study showed that the acetylcholinesterase (AchE) content was elevated considerably (*p* < *0.05*) in the Dox group by 5.98 times as linked to the oversight group. Quite the reverse, pretreatment with Lura (1 and 3 mg/kg) alleviated (*p* < *0.05*) AchE levels by 37.18% and 63.38%, respectively, as associated to the Dox group. Moreover, Dox administration resulted in an 86.54% reduction of dopamine (DA) level (*p* < *0.05*), as relevant to the oversight group. Contrarily, Lura (1 and 3 mg/kg) pretreatment demonstrated a significant elevation (*p* < *0.05*) of DA level by about 1.83 and 4.46-fold, respectively, in comparison with the Dox-treated rats (Fig. 7A, B). In addition, there was a significant increase (*p* < *0.05*) in cardiac troponin I (cTn-I) and creatine kinase-MB (CK-MB) serum levels in the Dox group, which amounted to 5.91 and 26.9-fold, respectively, when compared to the control group. In contrast, Lura (1 and 3 mg/kg) pretreatment showed off a meaningful diminishing (*p* < *0.05*) effect on these levels by about 41.94%, 68.21%, 39.29%, and 69.48%, respectively, related to the Dox group (Fig. 7C, D).

### Effect of Lura (1 and 3 mg/kg) on oxidative stress biomarkers in brain and cardiac tissues

**Fig. 8:**
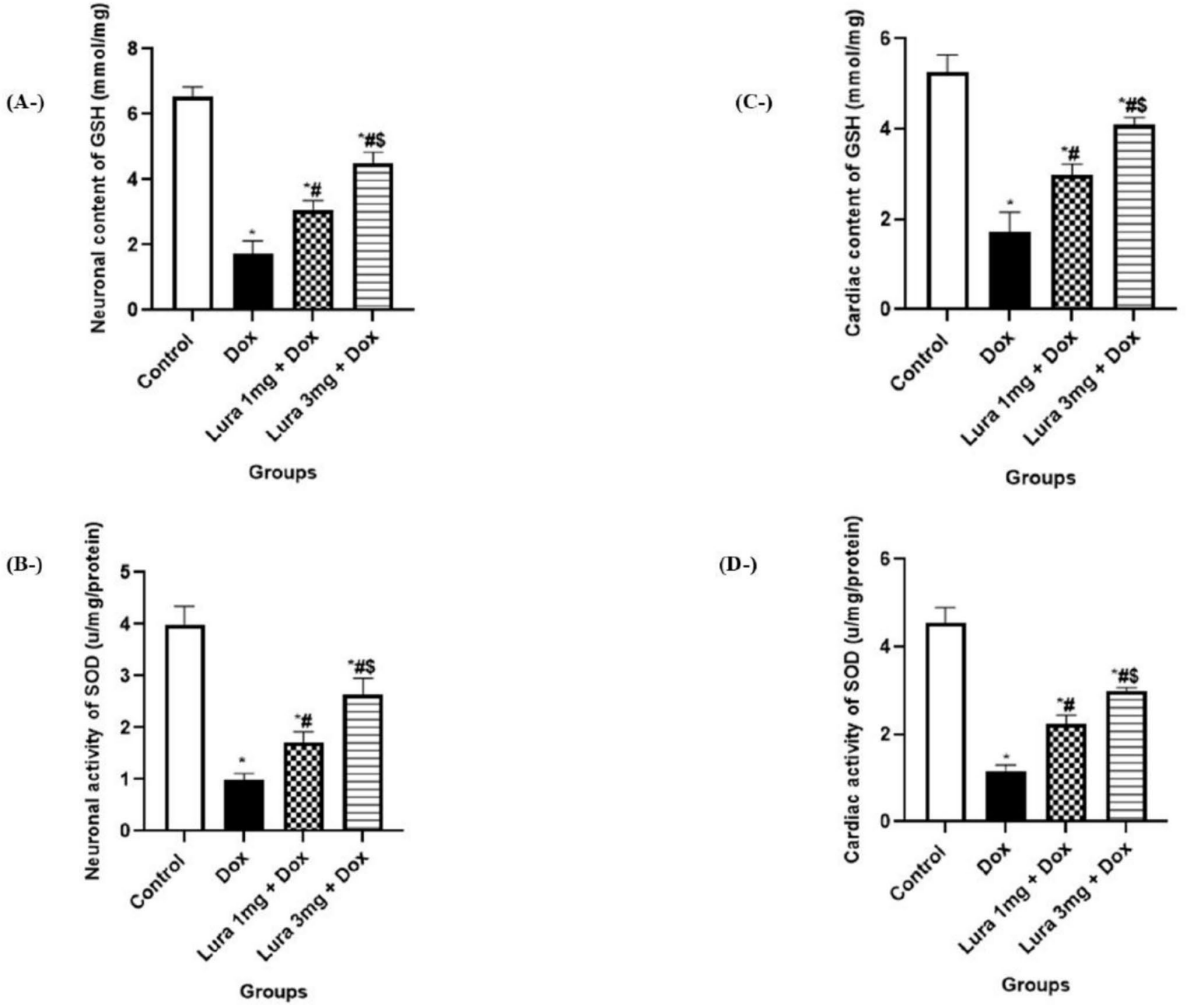
Effect of Lura (1 and 3 mg/kg) on neuronal and cardiac oxidative stress markers in Dox-treated rats. Neuronal markers. **A** GSH content. **B** SOD activity, cardiac markers. **C** GSH content. **D** SOD activity. The outcomes have been reported by way of mean ± SD (*n* = 6). One-way ANOVA was used to test significance, ensued by Tukey–Kramer multiple comparisons. *: conveys statistical disparity from the control group; #: conveys statistical disparity from the Dox group; $: conveys statistical disparity from the Lura 1 mg + Dox group, at *p* < *0.05*. Dox: doxorubicin; Lura: lurasidone; GSH: glutathione; SOD: superoxide dismutase

The outcomes of the existing study revealed that Dox-administered rats revealed a noteworthy decrease (*p* < *0.05*) in the content of GSH in brain besides heart tissue by 73.26% and 67.45%, respectively, along with a remarkable decline (*p* < *0.05*) in the SOD activity by 75.48% and 74.28%, respectively as related to the control group. Contrarily, pre-administered with Lura (1 and 3 mg/kg) significantly heightened (*p* < *0.05*) the level of GSHin neuronal tissue by 74.67% and 157.24% and cardiac tissue by 74.22% and 138.30%, respectively and notably elevated (*p* < *0.05*) SOD activity in neuronal tissue by 73.67% and 169.08% and cardiac tissue by 90.66% and 155.99%, respectively in comparison with Dox-administered group (Fig. 8A–D).

### Impression of Lura (1 and 3 mg/kg) on inflammatory markers in the brain and cardiac tissues

**Fig. 9:**
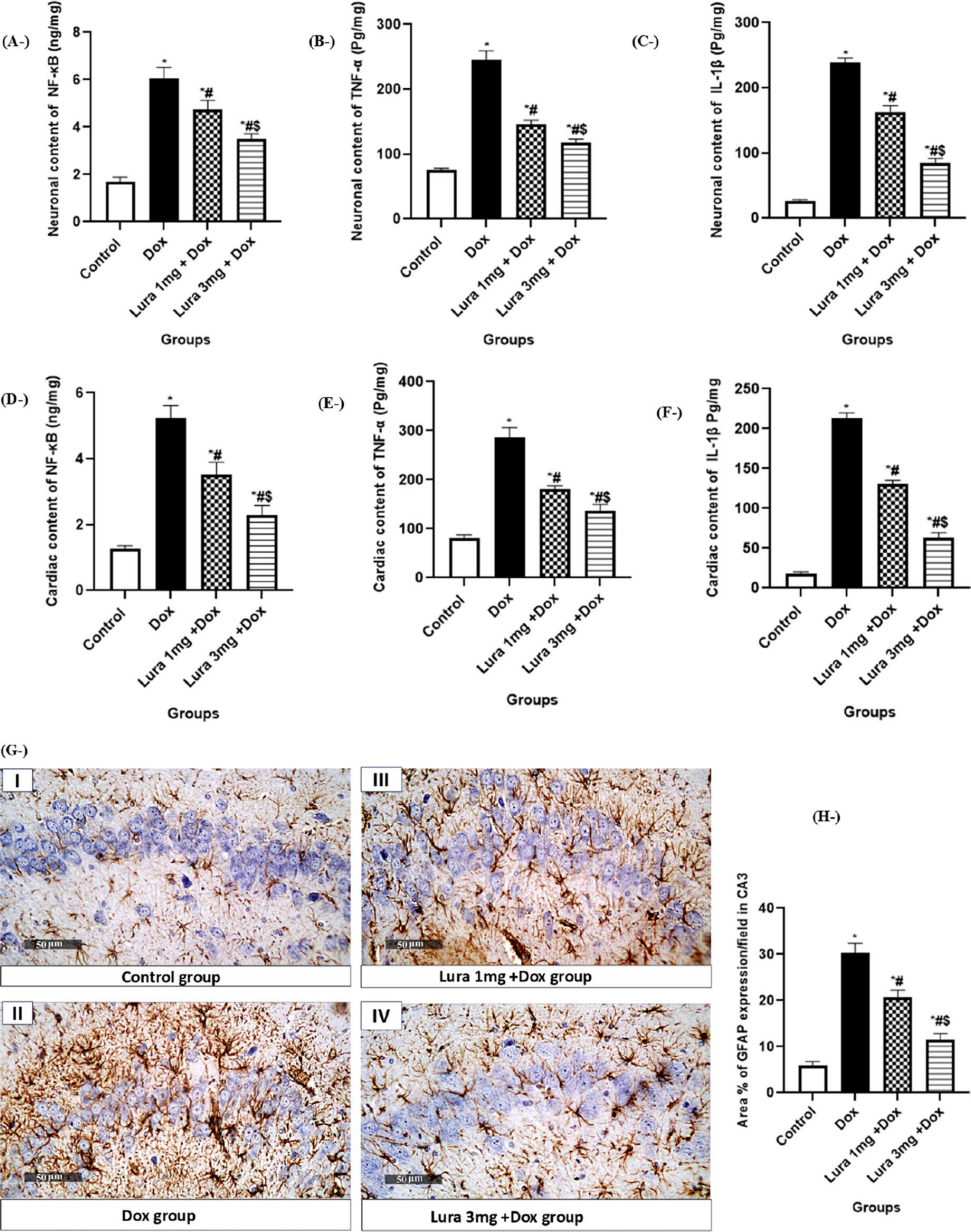

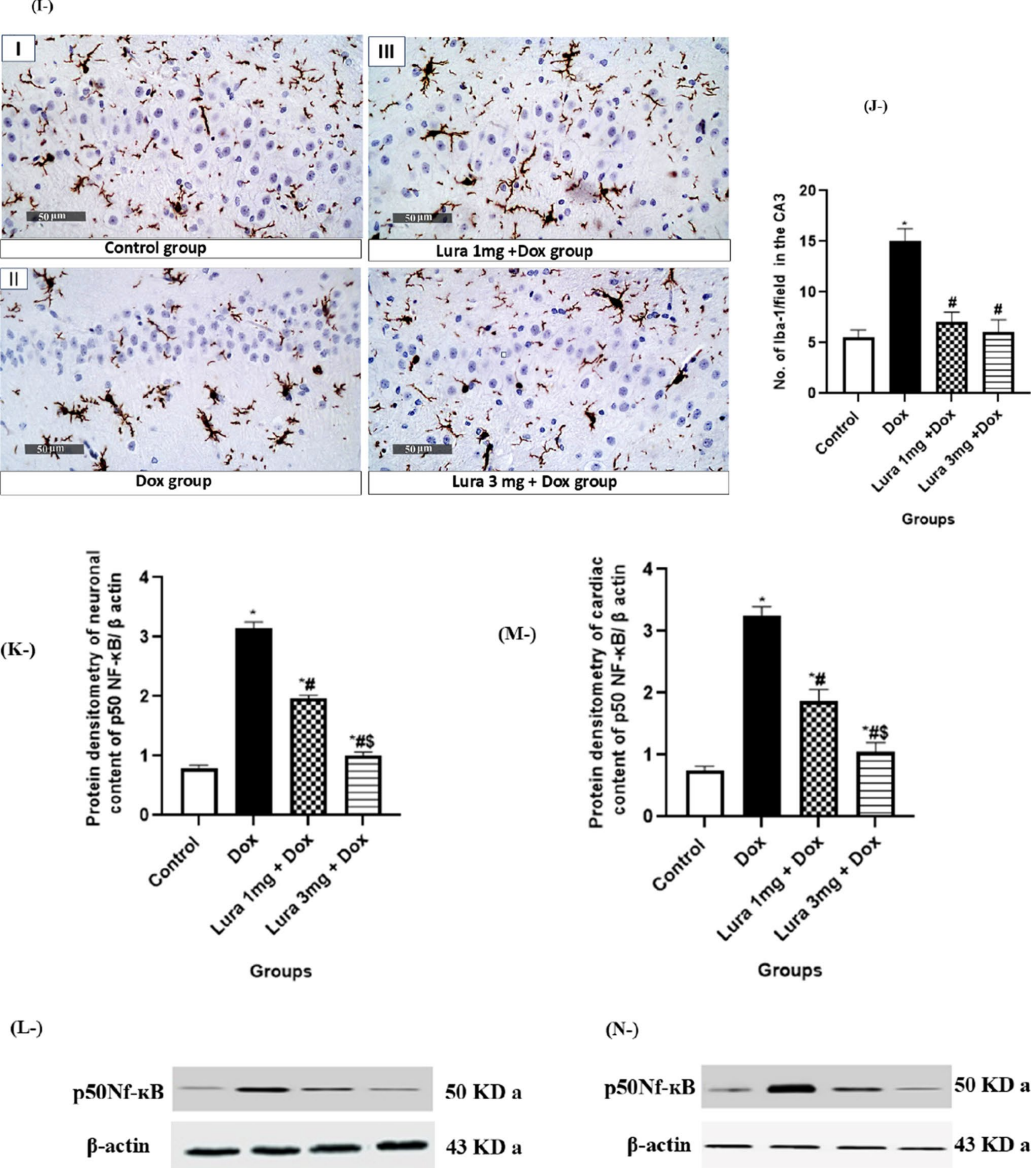
Effect of Lura (1 and 3 mg/kg) on neuronal and cardiac inflammatory markers in Dox-treated rats. Neuronal markers: NF-κB (**A**), TNF-α (**B**), and IL-1β (**D**); cardiac markers: NF-κB (**D**), TNF-α (**E**), and IL-1β (**F**) levels. **G**, **I** Representative photomicrography displays expression of neuronal GFAP and Iba- in the hippocampal region (CA3), respectively, in different groups. **H**, **J** Area % of GFAP expression and number of Iba-1 positive cells in the hippocampus (CA3 region), respectively (scale bars = 50 μm). **K**, **M** p50 subunit of NF-ĸB expression in neuronal and cardiac tissue, respectively. **L**, **N** Representative western blot images of p50 subunit of NF-ĸB expression in neuronal and cardiac tissue, respectively. The outcomes have been reported by way of mean ± SD (*n* = 6). One-way ANOVA was used to check significance, followed by Tukey–Kramer multiple comparisons. *: conveys statistical disparity from the oversight group; #: conveys statistical disparity from the Dox group; $: conveys statistical disparity from the Lura 1 mg + Dox group, at *p* < *0.05*. Dox: doxorubicin; Lura: lurasidone; NF-κB: nuclear factor kappa B; TNF-α: tumor necrosis factor-alpha; IL-1β: interleukin-1 beta; Iba-1: ionized calcium-binding adaptor molecule-1; GFAP: glial fibrillary acidic protein

As depicted in Fig. 9A–F, Dox induced a noteworthy increase (*p* < *0.05*) in the neuronal NF-κB, TNF-α, and IL-1β levels by 2.57-, 2.23-, and 8.11-fold, respectively, relative to the control group. Conversely, pretreatment with Lura (1 and 3 mg/kg) notably diminished (*p* < *0.05*) the levels of NF-κB by 21.85% and 42.11%, TNF-α by 40.33% and 51.73%, and IL-1β by 31.38% and 64.37%, respectively, relative to Dox-administered rats (Fig. 9A, B, C).

In a comparable context, Dox extensively enhanced (*p* < *0.05*) Iba1 expression in the hippocampus (CA3 region) by 172.7% compared with the control group. Interestingly, Lura (1 and 3 mg/kg) pretreatment markedly decreased (*p* < *0.05*) Iba-1 expression by 54.4% and 58.8%, respectively, compared with the Dox group. Moreover, there was a notable increase (*p* < *0.05*) in the expression of hippocampal (CA3) GFAP in the Dox-administered animals, which was approximately fourfold compared with the control. Contrarily, Lura (1 and 3 mg/kg) remarkably decreased (*p* < *0.05*) GFAP expression by 31.5% and 61.4%, respectively, compared with the Dox group (Fig. 9G–J).

As regards the heart tissues, administration of Dox resulted in a remarkable augmentation (*p* < *0.05*) in NF-κB, TNF-α, and IL-1β levels of about 3.14-, 2.53-, and 10.83-fold, respectively, compared with the control group. Contrarily, Lura (1 and 3 mg/kg) exhibited a considerable decrease (*p* < *0.05*) in the contents of NF-κB by 32.33% and 56.29%, TNF-α by 36.93% and 52.13%, and IL-1β by 38.58% and 70.53%, respectively, compared with the Dox group (Fig. 9D, E, F).

Moreover, it was found that Dox administration decisively boosted (*p* < *0.05*) the expression of p50 NF-κB in neuronal and cardiac tissues by 2.93- and 3.40-fold, respectively. Interestingly, pretreatment with Lura (1 and 3 mg/kg) considerably reduced (*p* < *0.05*) expression levels of p50 NF-κB in neuronal tissue by 37.52% and 68%, respectively, and in cardiac tissue by 42.54% and 68.09%, respectively, relative to the Dox-treated group (Fig. 9K–N).

### Impact of Lura (1 and 3 mg/kg) on neuronal and cardiac caspase-3 content and the number of intact neurons

**Fig. 10:**
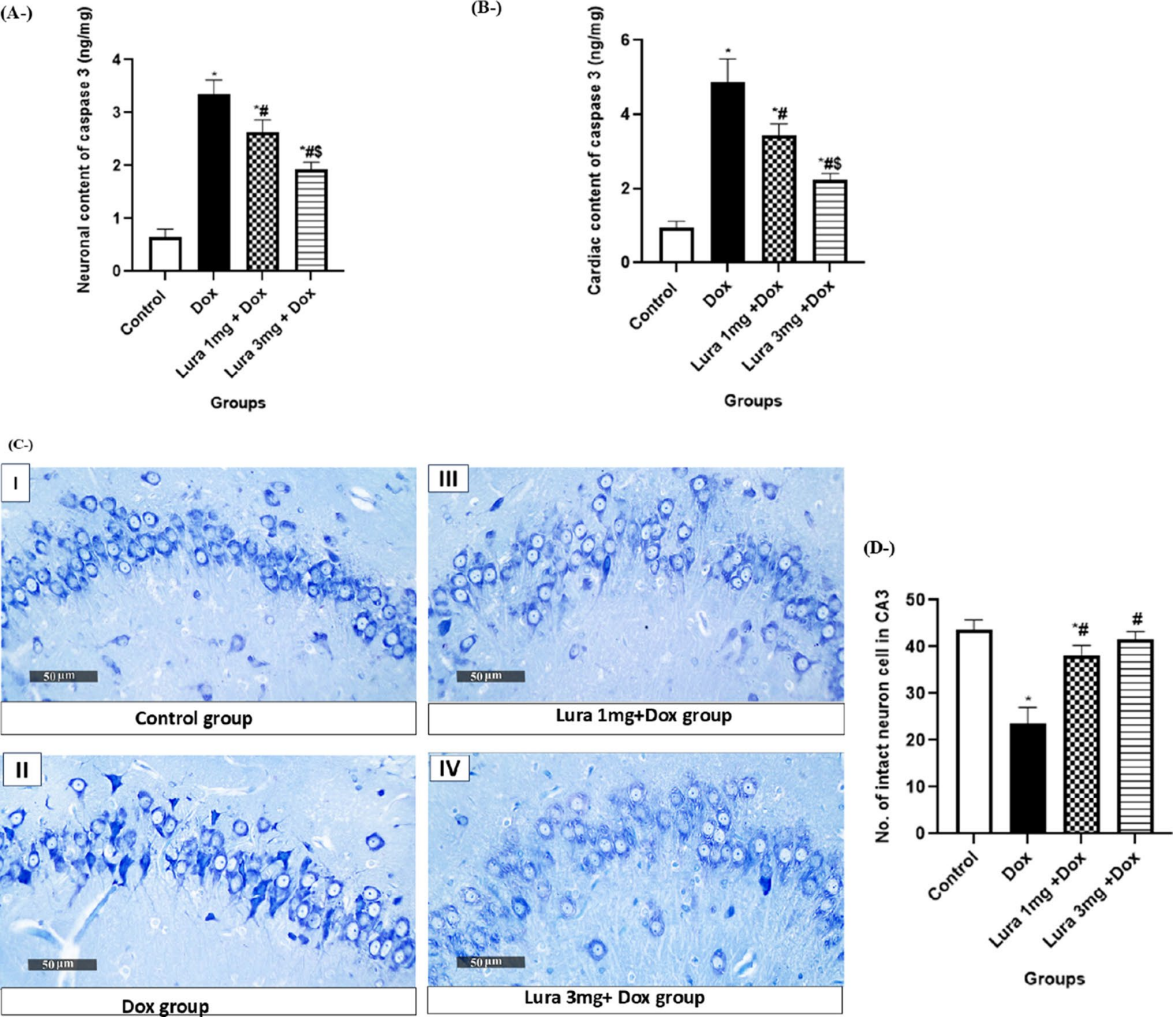

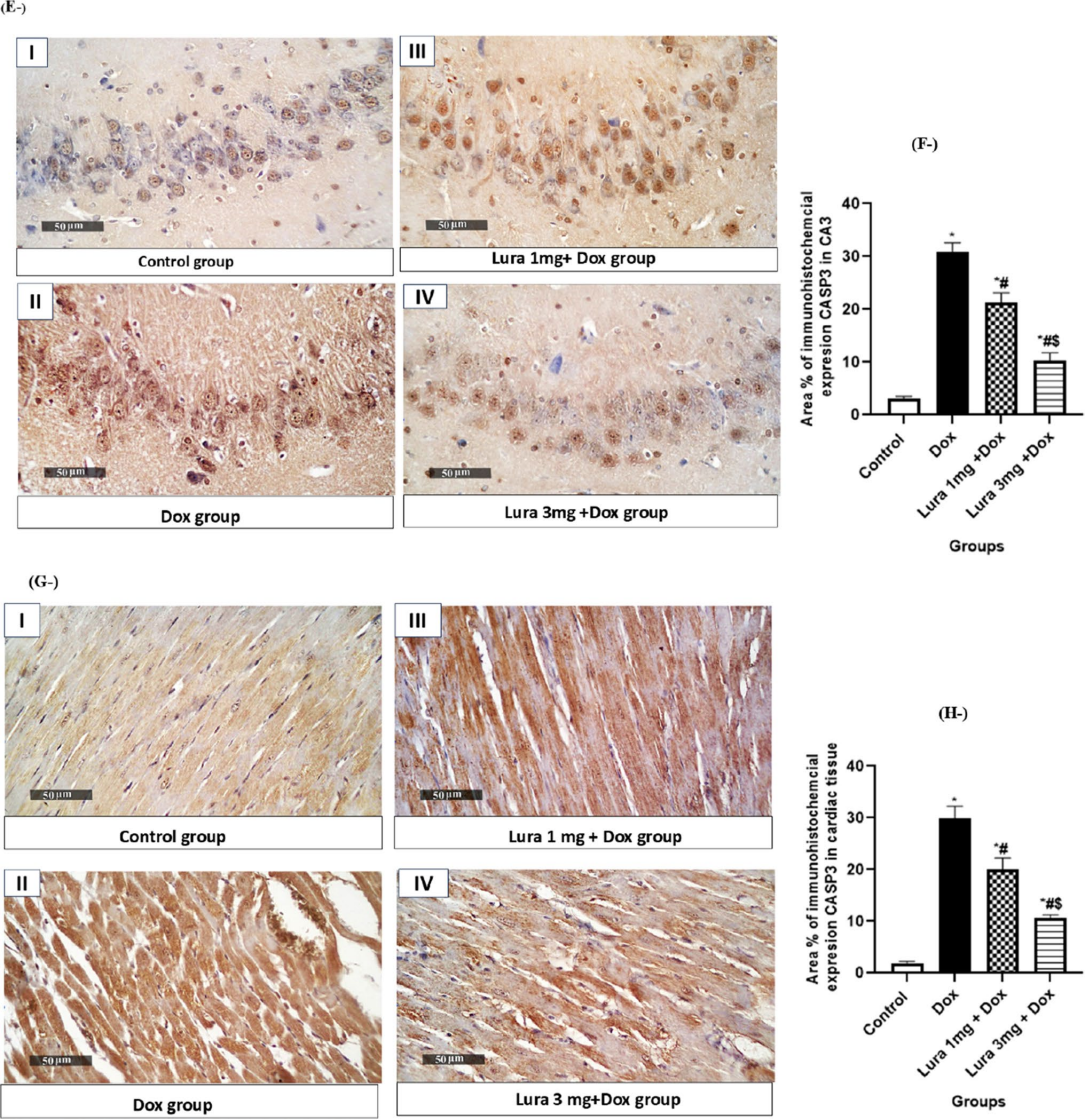
Effect of Lura (1 and 3 mg/kg) on neuronal and cardiac Casp 3 levels in rats and on the number of intact neuron cells in Dox-treated rats. Neuronal Casp 3 content (**A**), cardiac Casp 3 content (**B**), number of intact neuron cells in the CA3 region (**D**), % of area of cleaved Casp 3 expression in the CA3 region (F), and % of area of cleaved Casp 3 expression in cardiac tissue (**H**). Representative photomicrographs of brain and heart sections displaying the number of intact neurons in the CA3 region after staining with toluidine blue (**C**), cleaved Casp 3 expression in the CA3 region (E), and cleaved Casp 3 expression in cardiac tissue (**G**) (scale bars = 50 μm). The outcomes have been reported by way of mean ± SD (*n* = 6). One-way ANOVA was applied to check significance, followed by Tukey–Kramer multiple comparisons. *: conveys statistical disparity from the control group; #: conveys statistical disparity from the Dox group; $: conveys statistical disparity from the Lura 1 mg + Dox group, at *p* < *0.05*. Dox: doxorubicin; Lura: lurasidone; Casp 3: caspase 3

As elucidated in Fig. 10, treatment with Dox caused a significant augmentation (*p* < *0.05*) in the neuronal and cardiac levels of caspase 3 by 4.1- and 4.11-fold, respectively, compared with the control group. On the contrary, pretreatment with Lura (1 and 3 mg/kg) obviously reduced (*p* < *0.05*) neuronal caspase 3 content by 21.46% and 42.19% and cardiac caspase 3 content by 29.34% and 54.20%, respectively, compared with the Dox-treated group (Fig. 10A, B). Moreover, Dox-treated rats showed a significant reduction (*p* < *0.05*) in the number of intact neuron cells in the hippocampal region (CA3) by 45% compared with the oversight group. Alternatively, pretreatment with Lura (1 and 3 mg/kg) resulted in a remarkable increment (*p* < *0.05*) in the number of intact neuron cells compared with the Dox set by 60.2% and 73.4%, respectively (Fig. 10C, D).

Dox treatment triggered (*p* < *0.05*) the expression of cleaved Casp3 in the CA3 region and heart by approximately 9.5 and 10 times, respectively, compared to the oversight group. However, co-treatment with Lura (1 and 3 mg/kg) meaningfully mitigated (*p* < *0.05*) the expression of cleaved Casp3 by about 31.15% and 66.8% in the CA3 region, respectively, and 33% and 64.48% in cardiomyocytes relative to the Dox-treated rats, respectively (Fig. 10E–H).

### Influence of Lura (1 and 3 mg/kg) on the BDNF/TrkB/PI3K/Akt/CREB signalling pathway in brain and cardiac tissues

**Fig. 11:**
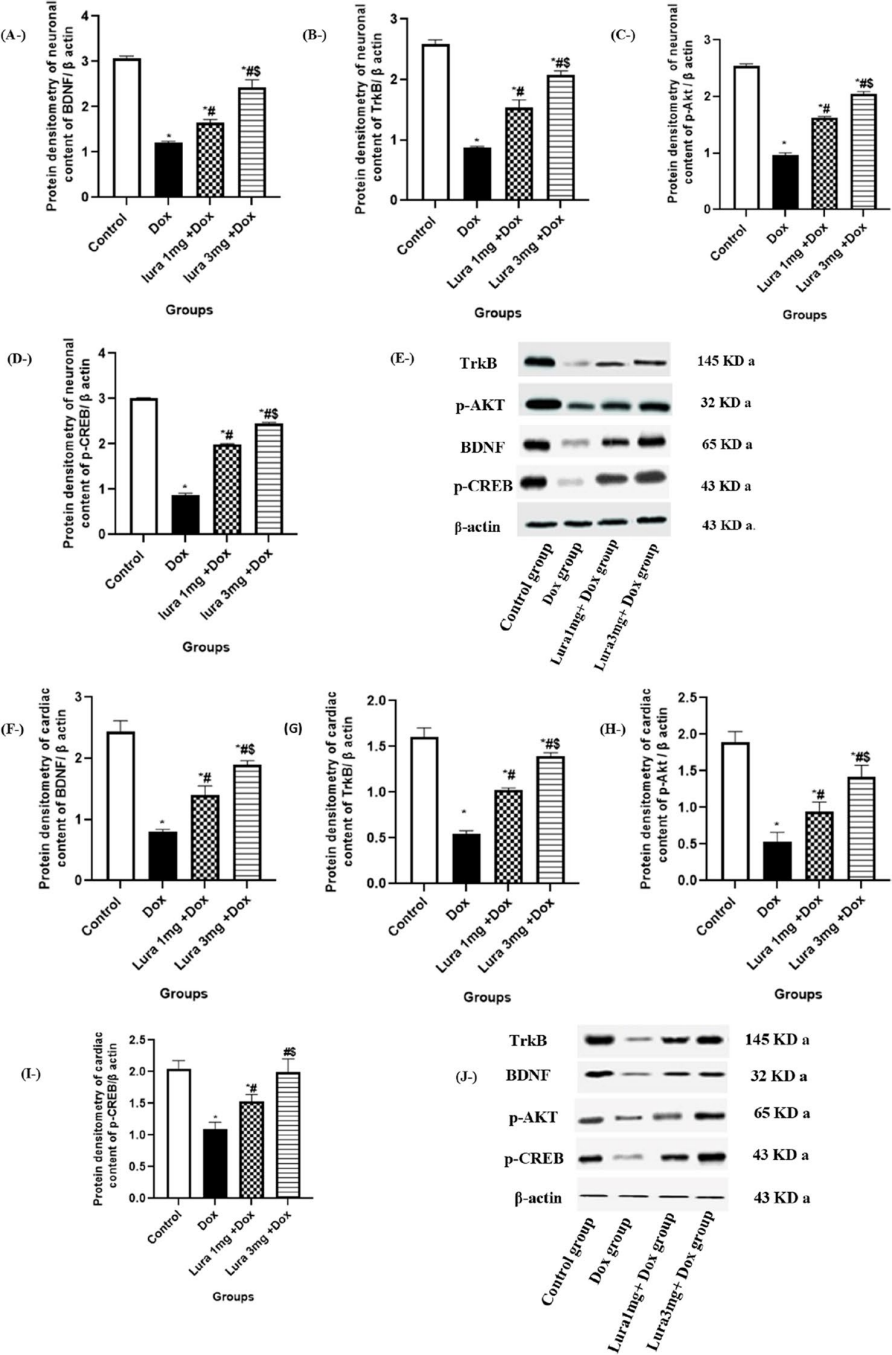

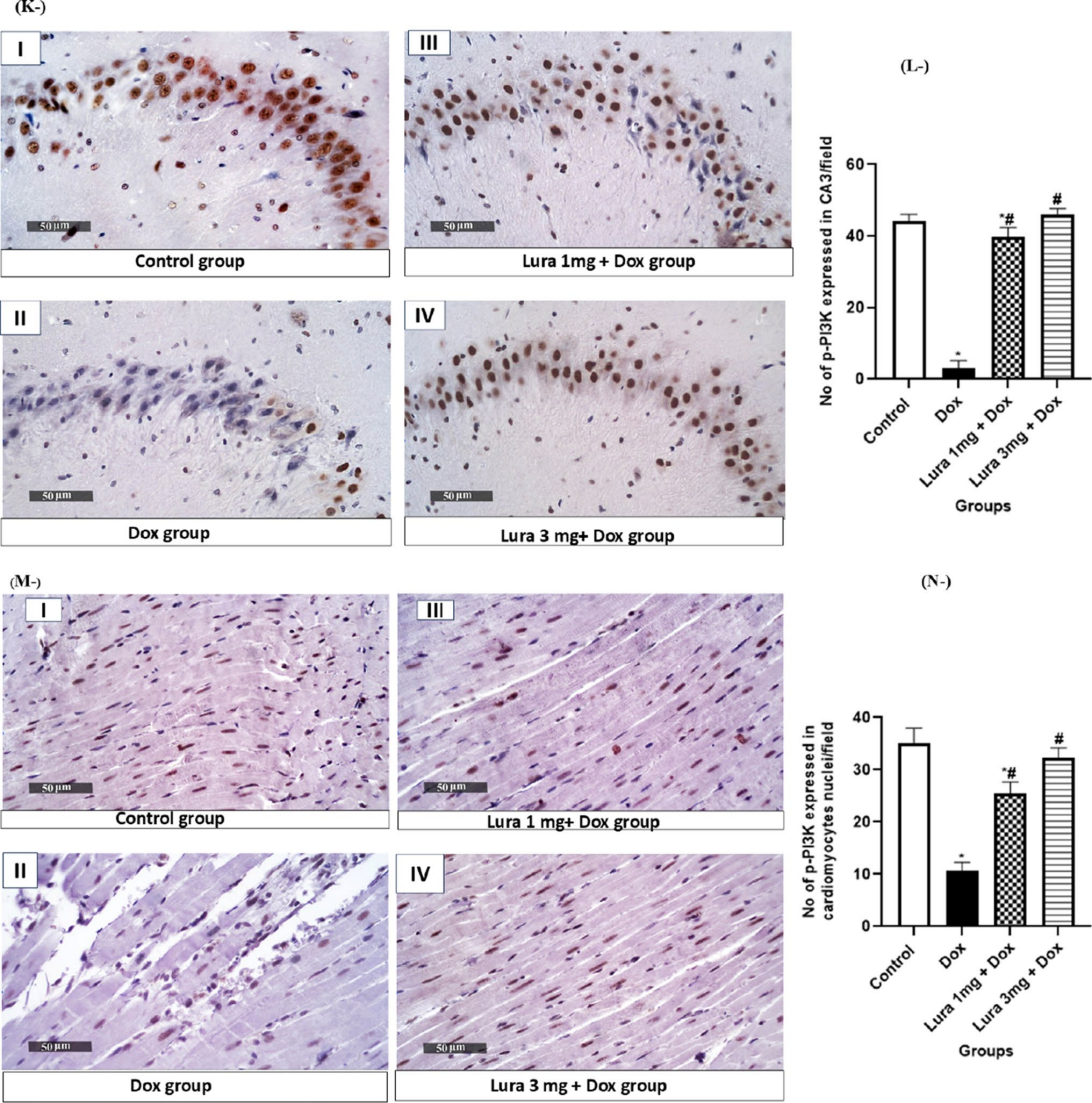
The effect of Lura (1 and 3 mg/kg) on neuronal and cardiac expression of the BDNF, TrkB, PI3K, Akt, and p-CREB protein levels in Dox-treated rats. Neuronal expression of BDNF (**A**), TrkB (**B**), p-Akt (**C**), p-CREB (**D**), p-PI3k (**L**); cardiac expression of BDNF (**F**), TrkB (**G**), p-Akt (**H**), p-CREB (**I**), p-PI3k expression (**N**) levels. Representative western blot images of neuronal and cardiac contents of TrkB, BDNF, p-Akt, and p-CREB proteins, respectively, (**E**, **J**), and representative photomicrographs displaying hippocampal (CA3) and cardiac expression of p-PI3K, respectively (**K**, **M**). The outcomes have been reported by way of mean ± SD (*n* = 6). One-way ANOVA was employed to check significance, ensued by Tukey–Kramer multiple comparisons. *: conveys statistical disparity from the oversight group; #: conveys statistical disparity from the Dox group; $: conveys statistical disparity from the Lura 1 mg + Dox group, at *p* < *0.05*. Dox: doxorubicin; Lura: lurasidone; BDNF: brain-derived neurotrophic factor; TrkB: tropomyosin receptor kinase B (TrkB); PI3K: phosphoinositide 3-kinase; p-Akt: phosphorylated protein kinase B (p-AKT); p-CREB: phosphorylated cAMP-response element binding protein; β actin: beta-actin

As illustrated in Fig. 11, Dox significantly downregulated (*p* < *0.05*) BDNF and TrkB protein expression, which were reduced compared with the control group in the brain by 60.32% and 66.22% and in the heart tissue by 67.15% and 66%, respectively. In contrast, Lura (1 and 3 mg/kg) administration resulted in a marked elevation (*p* < *0.05*) in both biomarkers, BDNF and TrkB, by about 35.14%, 100.82% and 75.83%, 137.91%, respectively, in the brain, and by 74.71%, 135.99%, and 87.15%, 155.96%, respectively, in the heart tissue.

In relevance to the control group, Dox-treated rats exhibited a meaningful decrease (*p* < *0.05*) in hippocampal (CA3) and cardiac expression of PI3K by 93.23% and 299.83%, respectively, while groups treated with lurasidone (1 mg and 3 mg) showed upregulated PI3K expression by 12.22-fold and 14.38-fold in neuronal tissues and by 139.21% and 203.28% in the myocardium, respectively, in comparison with Dox-treated rats.

Additionally, p-Akt expression was remarkably decreased (*p* < *0.05*) in brain and heart tissues by 62% and 71.70%, respectively, later the administration of Dox associated to the control group. In contrast, Lura (1 and 3 mg/kg) pretreatment significantly upregulated (*p* < *0.05*) the expression of p-Akt by 68.91% and 112.74% respectively, in the brain and by 75.93% and 164.55% respectively, in the heart, as related to the Dox group.

Moreover, the current study revealed a notable depletion (*p* < *0.05*) of the neuronal and cardiac p-CREB expression in Dox-treated rats by 70.82% and 46.38%, respectively, relative to the control. Nevertheless, pretreatment with Lura (1 and 3 mg/kg) caused a pronounced elevation (*p* < *0.05*) in the phosphorylation of CREB by 126.25% and 179.1%, respectively, in the brain and by 39.79% and 81.05%, respectively, in the cardiac tissue compared to the Dox-administered group (Fig. 11A–N).

### Effect of Lura (1 and 3 mg/kg) on neuronal and cardiac expression of mir34-a-5p and PGC-1α in Dox-treated rats

**Fig. 12:**
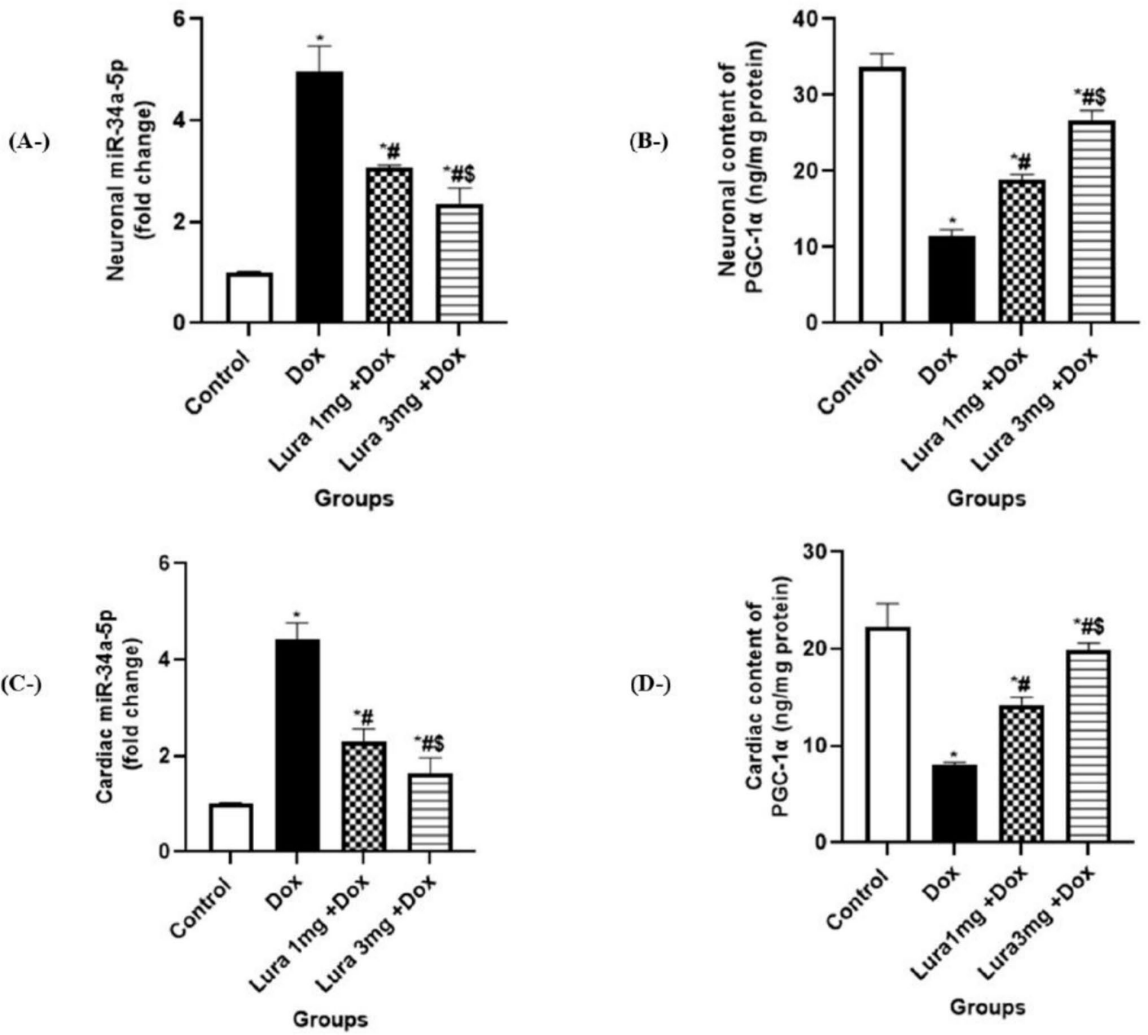
Effect of Lura (1 and 3 mg/kg) on neuronal and cardiac mir-34a-5p and PCG-1α in Dox-treated rats. Neuronal: mir-34a-5p (**A**), PGC-1α (**B**) relative expression, cardiac; mir-34a-5p (**C**), PGC-1α (**D**) relative expression. The outcomes are reported as mean ± SD (*n* = 6). One-way ANOVA was used to assess significance, followed by Tukey–Kramer multiple comparisons. *: conveys statistical disparity from the control group; #: conveys statistical disparity from the Dox group; $: conveys statistical disparity from the Lura 1 mg + Dox group, at *P* < *0.05*. Dox: doxorubicin; Lura: lurasidone; PCG-1α: peroxisome proliferator-activated receptor-gamma coactivator (PGC)−1alpha

As displayed in Fig. 12, the results of the RT-qPCR assay confirmed the significant upregulation (*p* < *0.05*) of neuronal and cardiac miR-34a-5p in the Dox group by 395% and 341%, respectively, as compared to the control group. However, miR-34a-5p expression was remarkably mitigated (*p* < *0.05*) in rats treated with Lura (1 and 3 mg/kg) by 38.18% and 52.32% in the brain and 48.07% and 63.03% in myocardium, respectively, as compared to the Dox group. Also, the Dox group showed a significant decrease (*p* < *0.05*) in PGC1ɑ by 66.22% in the brain and 63.76% in the heart tissue, in comparison with the control group, Contrarily, Lura groups (1 and 3 mg/kg) elucidated a remarkable upregulation (*p* < *0.05*) of PGC1ɑ by 65.78 and 133.77% in the brain and 75.96%, 145.35% in cardiac tissue, respectively, as related to the Dox set, Fig. 12A–D.

### Effect of Lura (1 and 3 mg/kg) on histopathological alterations in the hippocampal region (CA3 region) and the cardiac tissue in Dox-treated rats

**Fig. 13:**
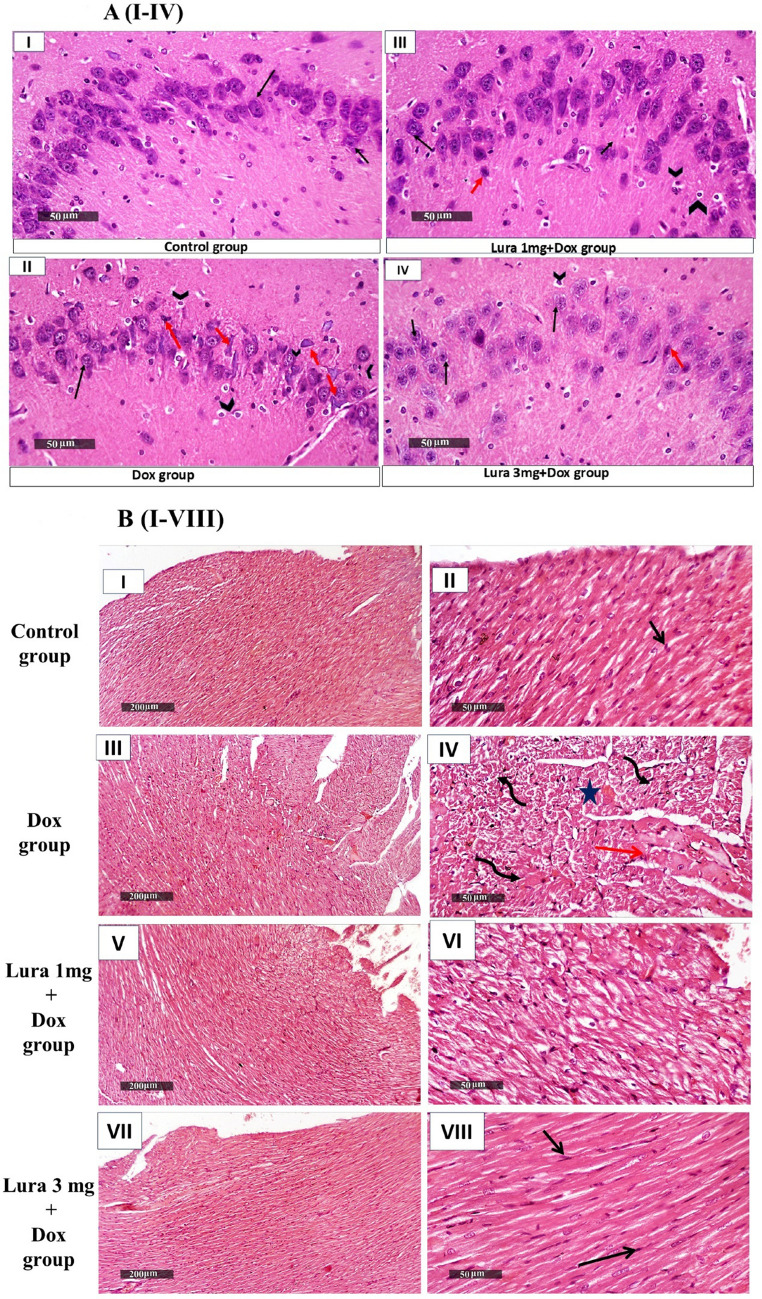
Representative microscopic photomicrographs of the brain and heart sections stained with H and E revealing the effect of Lura (1 and 3 mg/kg) on brain and heart tissues in Dox-treated rats. **A** Brain, scale bars; (I), (II), (III), (IV) = 50 µm. (I) The Control group exhibited intact neurons in the hippocampal (CA3) regions; (II) the Dox group showed significant damage in the neuronal cells accompanied by significantly higher scores of reactive microglial cells as well as astrocytic infiltrates; (III, IV) Lura (1 and 3 mg/kg) ameliorated neurodegeneration in most of the tissue sections (red arrow), with lesser scores of reactive glial cell infiltrates (arrowhead). **B** Heart tissue, scale bars; (I), (III), (V), (VII) = 200 µm, (II), (IV), (VI), (VIII) = 50 µm. (I, II) Control group showed a normal histological structure of the myocardium; (III, IV) Dox group exhibited significant histopathological variations represented by myocardial vacuolation and necrosis with marked thickening of the wall of coronaries; (V, VI) Lura 1 mg + Dox group revealed a moderate improvement, with a variable detection of degenerated and necrotic cardiac myofibers accompanied by mild peri-vascular oedema and vacuolation of cardiomyocytes; (VII, VIII) Lura 3 mg + Dox group elucidated marked improvement, comparable to the control group. Dox: doxorubicin; Lura: lurasidone; H and E: hematoxylin and eosin

In the present study, histopathological examination revealed healthy neurons in the hippocampus (CA3), with no alterations detected in the control group (Fig. 13A–I). Meanwhile, examination of the Dox group showed multiple, widespread necrotic pyramidal neurons (red arrow) scattered among apparently intact cells (black arrow)(Fig. 13A–II). Also, microglial processes correlated with neuroimmunological response features showed a significantly greater presence of reactive microglial cells as well as astrocytic infiltrates (arrowhead). A moderate neuroprotective effect was detected in the Lura 1 mg + Dox group (black arrow), with minimal neuronal damage in most tissue sections (red arrow) and fewer reactive glial cell infiltrates (arrowhead), with the brain matrix appearing normal (Fig. 13A–III). The Lura 3 mg + Dox group demonstrated the same neuronal protective efficacy as the low-dose samples (Lura 1 mg + Dox). Furthermore, a few infiltrates of reactive glial cells (Fig. 13A–IV).

As for the heart tissue, the control group’s microscopic analysis (Fig. 9B–I) revealed normal, obvious flawless cardiac wall layer histological structures with nearly uninjured branched, organized cardiomyocytes in addition to intact subcellular structure (black arrow) and vasculatures free of aberrant infiltrative cells.

Meanwhile, the Dox group (Fig. 13B–II) exhibited marked histopathological changes represented by degeneration of cardiomyocytes in the subendocardial zone (spiral arrow) with focal scattered necrotic cardiomyocytes (red arrow), accompanied by many congested vasculatures (star). The histopathological findings showed that Lura (1 and 3 mg/kg) protected the cardiac cell architecture against the injurious effects of Dox; however, minimal congested BVs were observed (Fig. 13B–III, IV).

#### Effect of Lura (1 and 3 mg/kg) on the cytotoxic activity of Dox

**Fig. 14:**
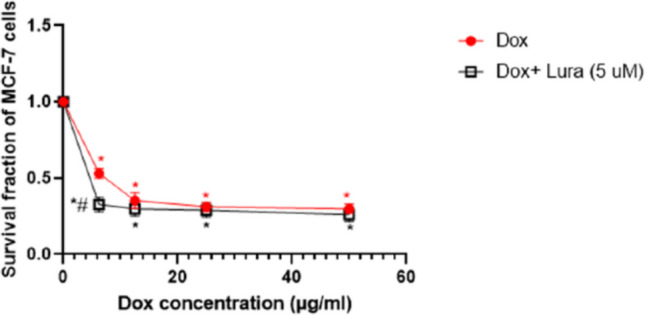
The effect of variable concentrations of Dox (5–50 μg/ml) solely or in combination with the IC_10_of Lura in the MCF-7 cell line. Results are expressed as mean ± SD of 3 independent experiments performed in triplicate. One-way ANOVA was used to test significance, followed by Tukey–Kramer multiple comparisons. *: Notably different from the control group; #: Notably different from Dox alone at *p* < *0.05* using two-way ANOVA followed by Tukey’s post hoc test for multiple comparisons. Dox: doxorubicin; Lura: lurasidone; IC_10_: half maximal inhibitory concentration

Figure 14 displays the cell viability, represented as the survival fraction, compared with control cells. Treatment of human breast (MCF-7) cancer cells with variable concentrations of Dox for 24 h remarkably decreased (*p* < *0.05*) the survival fraction of cells. Dox IC_50_ was found to be 7.5 µg/mL for MCF-7. In the same context, treatment of MCF-7 cells with IC_10_ of Lura (5 µg/mL for MCF-7) for 24 h before Dox addition did not affect the IC_50_ of Dox.

## Discussion

Despite the therapeutic efficacy of DOX against various cancer types, its use is almost inevitably accompanied by the development of cardiotoxicity and neurological adverse effects such as anxiety and depression (Renu and Arunachalam 2018; Aygun and Gul 2018).

Furthermore, it is well known that cardiac biomarkers such as cTnI and CK-MB are sensitive diagnostic markers used to assess myocardial toxicity (Kim et al. 2016). In our study, DOX augmented the serum levels of cTnI and CK-MB. Conversely, co-administration of Lura mitigated the alterations in the cardiac biomarkers, implying protection against Dox-induced cardiac toxicity.

Regarding the pathophysiological mechanism through which DOX triggers cardiotoxicity, oxidative stress remains the hallmark pathogenic pathway of DOX-induced cardiomyopathy, where an upsurge in ROS and a decrement in antioxidant levels take place with the subsequent activation of inflammatory cascades, leading to the generation of pro-inflammatory cytokines, such as TNFα and IL-1β (Sangweni et al. 2022; Shi et al. 2023). Likewise, DOX activates macrophages and monocytes by promoting the generation of TNF-α, which in turn activates the TNF receptor (TNFR), ultimately resulting in structural alterations and dilated cardiomyopathy (Yu et al. 2018). In the same context, Dox compromises the activity of Topoisomerase II beta (Top 2β), leading to DNA damage and transcriptional changes that result in ROS liberation and culminate in functional and structural damage to cardiomyocytes (Zhang et al. 2020).

In accordance with these outcomes, DOX administration remarkably diminished GSH levels and SOD enzyme activity and provoked IL-1β, NF-ĸB, and TNF-α levels in the cardiac tissue. The imbalance of redox homeostasis and aggravation of the production of the inflammatory cytokines were remarkably ameliorated by the two dose levels of Lura. In accordance with our study, the antioxidant properties of Lura were previously discussed (Spero et al. 2022).

Regarding the neurological complications, the present study showed that Dox provoked anxiety and depression-like behaviors in rats, as evidenced by the open-field test and the forced-swimming test, respectively, compared with the control group.

OFT has been broadly used in rats to appraise anxiety-like behaviors (Aygun and Gul 2018; Sun et al. 2019). One of the drawbacks of the FST is that short-range antidepressant handling reverses immobility, while, clinically, it can take weeks for the same antidepressants to elevate mood (as with many antidepressant-sensitive paradigms). Like many behavioral models, this test requires parallel locomotor activity assays to account for the influence of motor performance on behavior (Gencturk and Unal 2024).

The present study has revealed that DOX decreased locomotion activity and increased exploratory behaviors and grooming reactions compared to the control group. Interestingly, Lura hindered these behavioral abnormalities at both dose levels.

Moreover, the FST was selected as a behavioral measure of the depression-like state being tested, in which despair behavior has been labelled as a lack of mobility (Yankelevitch-Yahav et al. 2015). Furthermore, the FST is a behavioral analysis conducted on rodents to assess the effectiveness of novel compounds to induce or prevent depression-like states (Can et al. 2012). The current study revealed that the injection of Dox caused a noteworthy augmentation of immobility time and a marked reduction of swimming time compared with the control group. In contrast, Lura, at both dose levels, significantly reversed Dox-induced depression-like behavior.

Previous behavioral studies have asserted the antipsychotic and antidepressant activities of Lura in different animal models (Luoni et al. 2014), which have been shown to be due to the inhibitory effect of Lura on 5-HT7 receptors. Moreover, Lura boosted norepinephrine release via antagonism of α−2c receptors and activation of 5-HT1A and 5-HT2A receptors, resulting collectively in a surge in dopamine activity, particularly in the prefrontal cortex, leading finally to sustained, robust, and rapid anxiolytic and antidepressant effects (Okada et al. 2019; Goldberg et al. 2023).

In the same context, alterations in the levels of the neurotransmitter dopamine (DA) and of acetylcholinesterase (AChE) are mainstay players in the pathophysiology of neuroinflammation and neurodegeneration triggered by the administration of chemotherapeutic agents (Du et al. 2021; Mani et al. 2022). Indeed, cognitive and psychiatric disorders are modulated by AChE and DA levels in various brain regions (Antkiewicz-Michaluk et al. 2016).

AChE conveys neuroprotection against DOX-induced cognitive impairments through reducing brain oxidation, neuroinflammation, increasing neurogenesis, improving long-term plasticity (LTP), preventing glial cell activation, and inhibiting apoptosis (Khadrawy et al. 2021; Alsikhan et al. 2023). Moreover, it maintains the level of acetylcholine (ACh) in neurons, which improves neuronal function (Ongnok et al. 2021; Du et al. 2021; Mani et al. 2022). In the same regard, DA is essential for the cellular consolidation of hippocampal-dependent memories (O’Carroll et al. 2006; Pezze and Bast 2012).

Previous studies stated that Dox provoked oxidative stress and elevated ROS, which mediated alterations in neurotransmitter levels and resulted in irregular nerve signal transduction and subsequent changes in the activity of cholinergic and monoamines activity, culminating in cognitive deficits (Du et al. 2021; Mani et al. 2022).

In the current study, Dox enhanced AChE expression and distinctly reduced (DA) levels as compared to the control group. In contrast, co-treatment with Lura downregulated AChE expression and remarkably elevated DA compared to the Dox-treated group. This could be related to the binding affinity of Lura towards 5-HT7 receptors, resulting in outstanding monoaminergic and cholinergic enhancement.

Undoubtedly, oxidative stress as well as peripheral inflammation play a fundamental role in the induction of chemo-brain triggered by DOX (Lyu et al. 2021; Alsaud et al. 2023; Alsikhan et al. 2023). Despite not being capable of passing through the BBB (Ongnok et al. 2020; Du et al. 2021; Mani and Alshammeri 2023), Dox still has an impact on neuronal cells by eliciting peripheral over-expression of cytokines, where the produced inflammatory mediators disturb the tight junctions of the BBB, thus passing through, leading to the inciting of microglia and astrocytes, which in turn promote the massive production of cytokines for instance IL-1β and TNF-α (El-Agamy et al. 2018; Wang et al. 2021). Moreover, a previous study reported that Dox has direct neurotoxicity on the brain by crossing the BBB through the vascular-associated apical projection of neuronal stem cells and causing neuroinflammation (Du et al. 2021).

Astrocytes are the most plentiful and largest glial cells that support neuronal function in several ways, including homeostatic regulation and improvement in synaptic plasticity**,** while microglia are thought to have an important role in immunological responses to internal or external stimuli. Upon being compromised, astrocytes and microglia release considerable amounts of GFAP and Iba1, respectively (Jung et al. 2020; Dias-Carvalho et al. 2022).

The current investigation showed that following DOX treatment, there was a remarkable inhibition of antioxidant levels of SOD and GSH, along with increased levels of the pro-inflammatory cytokines NF-κB, IL-1β, and TNF-α in the brain. In the same regard, Dox treatment resulted in the overexpression of GFAP and Iba1 in the CA3 region and cortex, suggesting disruption of the integrity of astrocytes and microglial cells. Of interest, Lura pretreatment at both dose levels remarkably enhanced the antioxidant levels, attenuated neuroinflammation, and downregulated the expression of GFAP and Iba1 proteins.

Our study was further extended to explore the underlying neuro- and cardio-protective mechanisms of Lura. Several studies reported the detrimental role of BDNF in cell survival in both neurons (Van Kanegan et al. 2014) and the heart (Feng et al. 2015; Fulgenzi et al. 2015). The attachment of BDNF to its specific receptor TrKB triggers activation of PI3K, which in turn activates the Akt/CREB signaling route. The PI3K/Akt/CREB path is widely identified as promoting the survival, differentiation, and growth of neuronal and non-neuronal cells and preventing cellular apoptosis (Li et al. 2023). Also, previous investigations have revealed that activated AKT protects against cardiac injury induced by Dox administration (Liao et al. 2020; Zhang et al. 2020). Moreover, neuroprotection was conveyed through the PI3K/Akt/CREB signaling pathway (Luo et al. 2015; Hu et al. 2017). Reportedly, decreased phosphorylation of Akt/CREB was correlated with neuronal deficiency in neurodegenerative diseases in animal models. In contrast, accelerated Akt/CREB activity in the brain suppresses neuroinflammation and boosts neuronal function (Dong et al. 2018). Interestingly, the c-AMP-response element-binding protein (CREB) is a key regulator of BDNF production; phosphorylation increases BDNF expression (Li et al. 2018).

Our data showed that Lura was able to downregulate Dox-induced brain and cardiac toxicity by enhancing the transcription of BDNF, TrkB, PI3K, p-Akt, p-CREB proteins, and apoptotic caspase-3 in rat heart and brain tissues.

In the same context, Lura pretreatment downregulated the expression of miR 34a-5p in brain and heart tissues, with the consequent upregulation of PCG-1α, an essential modulator for the proper functioning of mitochondria and mitochondrial biogenesis. Previous studies reported that the liberation of reactive oxygen species causes upregulation of miR 34a-5p, which inhibits SIRT1 expression, leading finally to mitochondrial dysfunction (Payne et al. 2023; Zhou et al. 2024).

It is noteworthy that handling MCF-7 cancer cells with the IC_10_ of Lura for 24 h before addition of Dox did not affect Dox’s IC50.

## Conclusion

**Fig. 15:**
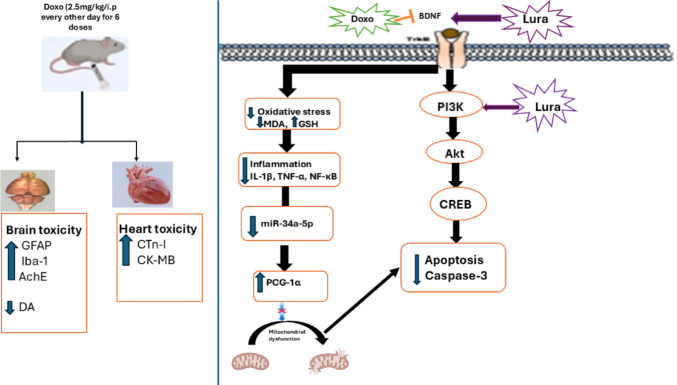
Summary of the cardio- and neuroprotective effect of Lurasidone in Dox-treated rats. Dox administration caused 1. Augmented oxidative stress 2. Aggravated inflammation which is evidenced by increased levels of NF-κB, TNF-α, and IL-Iβ 3. Enhanced apoptosis as confirmed by elevated caspase-3 level 4. Upregulated expression of miR-34a-5p and downregulation of PCG-1α. Lura alleviated the aforementioned injurious aspects partly through the enhancement of the BDNF/PI3K/TrkB/Akt/CREB signaling pathway, along with the downregulation of miR-34a-5p expression and augmented PCG-1α expression. Dox: doxorubicin; Lura: lurasidone; NF-κB: nuclear factor kappa B; TNF-α: tumor necrosis factor-alpha; IL-1β: interleukin-1 beta; BDNF: brain-derived neurotrophic factor; PI3K: phosphoinositide 3-kinase; TrkB: tropomyosin receptor kinase B (TrkB); p-Akt: phosphorylated protein kinase B; p-CREB: phosphorylated cAMP-response element binding protein; PCG-1α: peroxisome proliferator-activated receptor-gamma coactivator (PGC)−1alpha

In conclusion, our study revealed that Lura, a multi-receptor modulator, conferred cardioprotection and neuroprotection in Dox-treated rats by triggering stimulation of the BDNF/TrkB/PI3K pathway and the downstream Akt and CREB molecular signaling pathways, and hence alleviating oxidative stress, inflammation, and apoptosis. In the same context, Lura downregulated miR-34a-5p expression and thus boosted PCG-1α expression, enhancing mitochondrial function. It is important to note that our research is the first to correlate BDNF upregulation with cardio- and neuroprotection in Dox-treated rats (Fig. 15).

## Limitations of the study

While miR-34a-5p and PGC-1α showed coordinated changes in response to treatments, the study did not directly test whether miR-34a-5p functionally regulates PGC-1α in this model. The proposed miR-34a-5p/SIRT1/PGC-1α linkage is based on prior literature, and future mechanistic studies are needed to confirm this causal relationship.

Moreover, the present study is the prophylactic administration of Lura, which was initiated 7 days prior to doxorubicin exposure. This pre-treatment paradigm does not fully emulate clinically relevant therapeutic or concurrent scheduling. Accordingly, future investigations are warranted to determine whether Lura confers comparable protection when administered at time points aligned with standard chemotherapeutic protocols.

## Supplementary Information

Below is the link to the electronic supplementary material.

ESM 1 (PDF 227 KB)

ESM 2 (PDF 273 KB)

ESM 3 (XLSX 9.49 KB)

ESM 4 (XLSX 11.1 KB)

ESM 5 (XLSX 1.15 MB)

ESM 6 (XLSX 377 KB)

ESM 7 (XLSX 10.7 KB)

ESM 8 (XLSX 12.6 KB)

ESM 9 (XLSX 189 KB)

## Data Availability

All source data for this work (or generated in this study) are available upon reasonable request.
