# Supplementary material for: Repurposing lurasidone to alleviate doxorubicin-induced cardiotoxicity and neurotoxicity via BDNF/TrkB/PI3K/Akt/CREB and miR-34a-5p/PGC-1α pathways
Source: Naunyn Schmiedebergs Arch Pharmacol. 2026 Mar 31;399(9):13731–57. doi: 10.1007/s00210-026-05019-z (PMC13357379; doi:10.1007/s00210-026-05019-z)

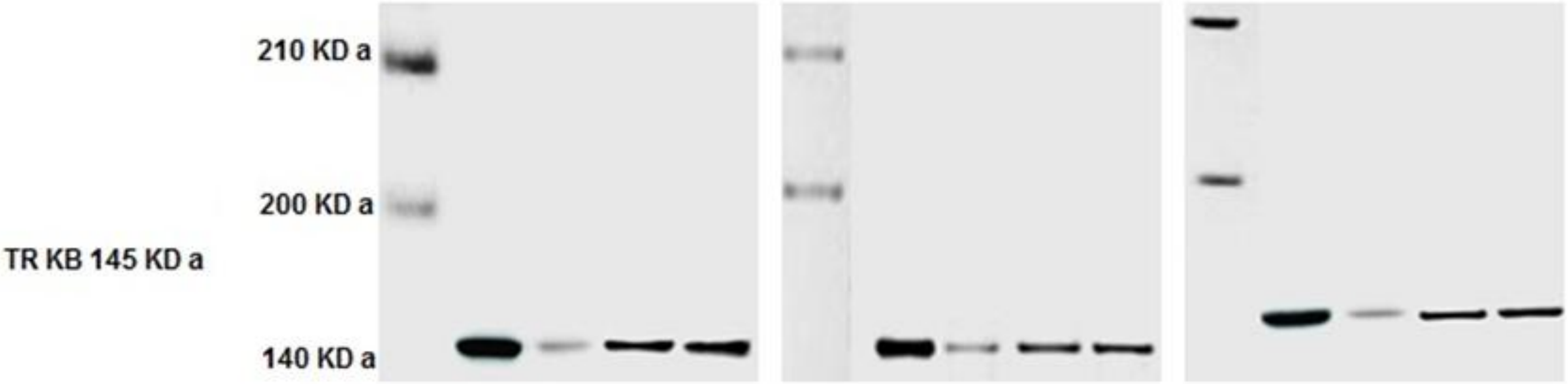
210 KD a
200 KD a
TR KB 145 KD a
140 KD a

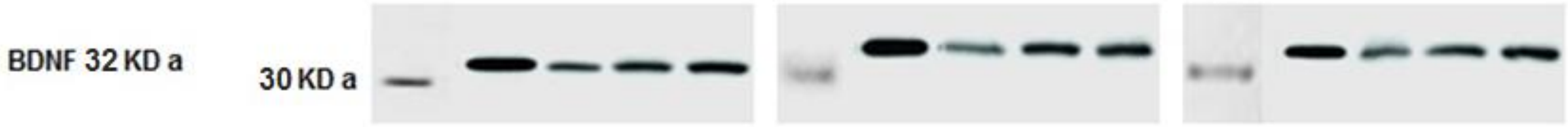
BDNF 32 KD a
30 KD a

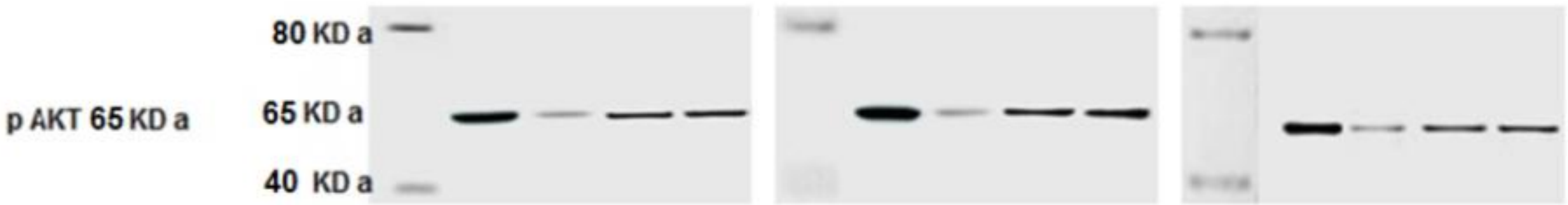
p AKT 65 KD a
80 KD a
65 KD a
40 KD a

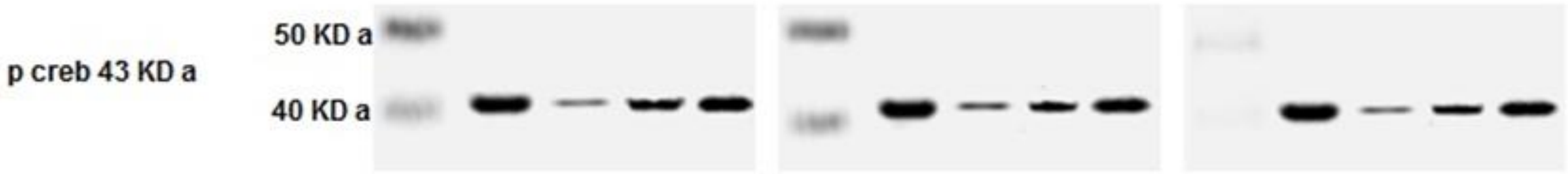
p creb 43 KD a
50 KD a
40 KD a

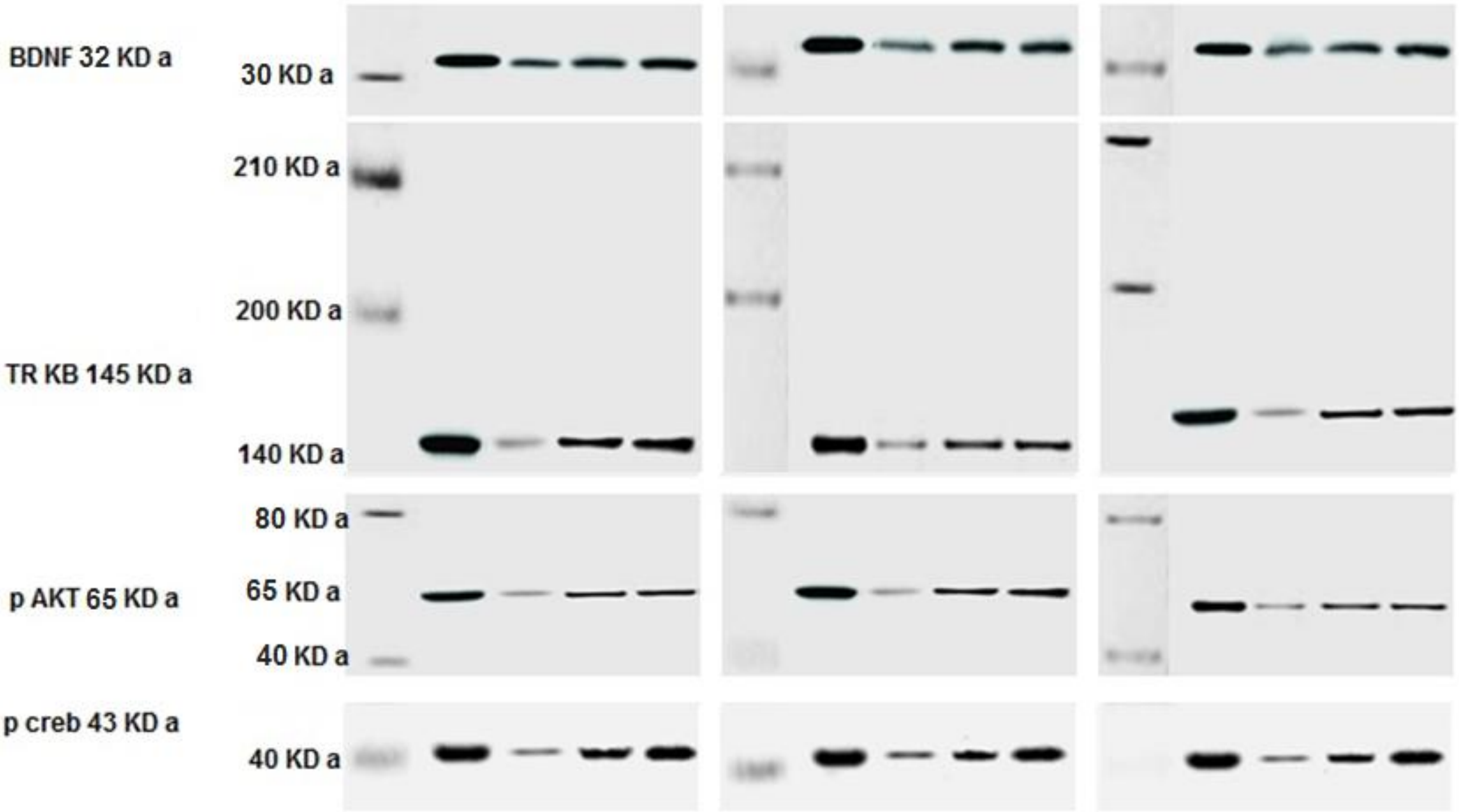
BDNF 32 KD a
30 KD a
210 KD a
200 KD a
TR KB 145 KD a
140 KD a
80 KD a
65 KD a
p AKT 65 KD a
40 KD a
p creb 43 KD a
40 KD a

Brain β- actin

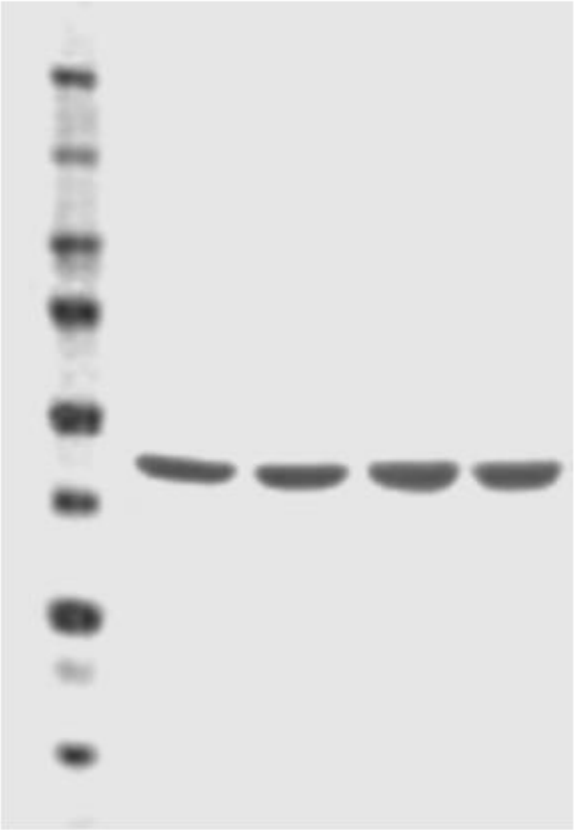

Brain β- actin

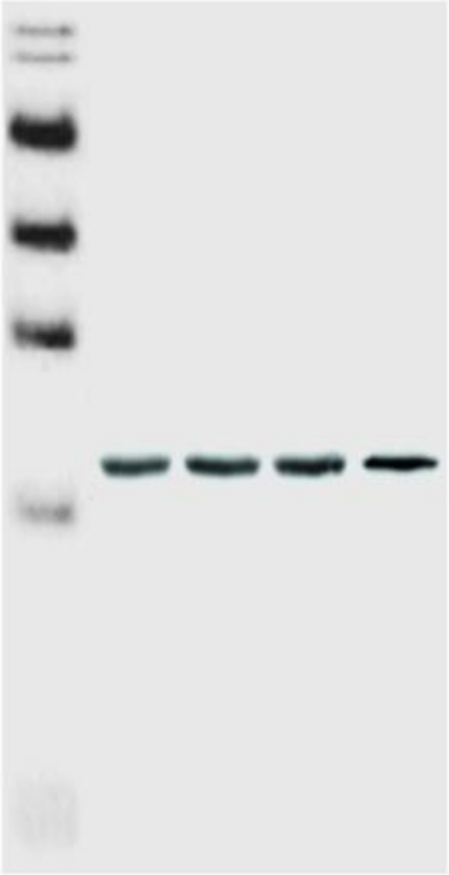

Brain β- actin

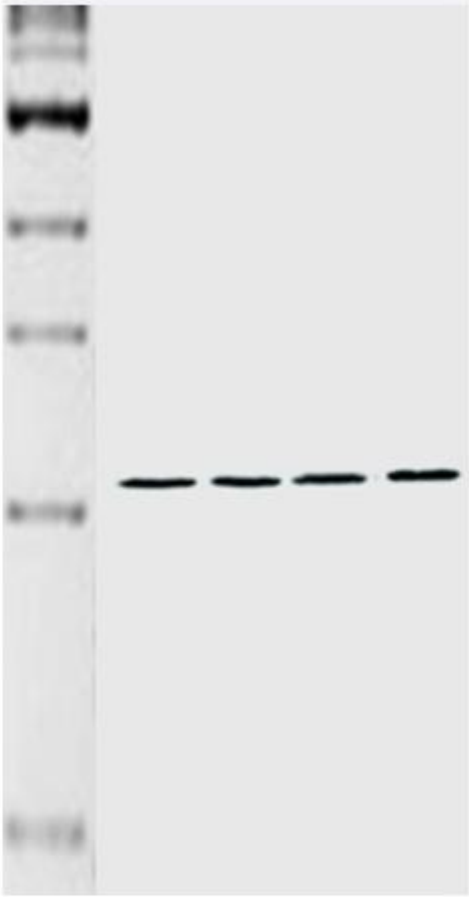

Brain NF-κB p50

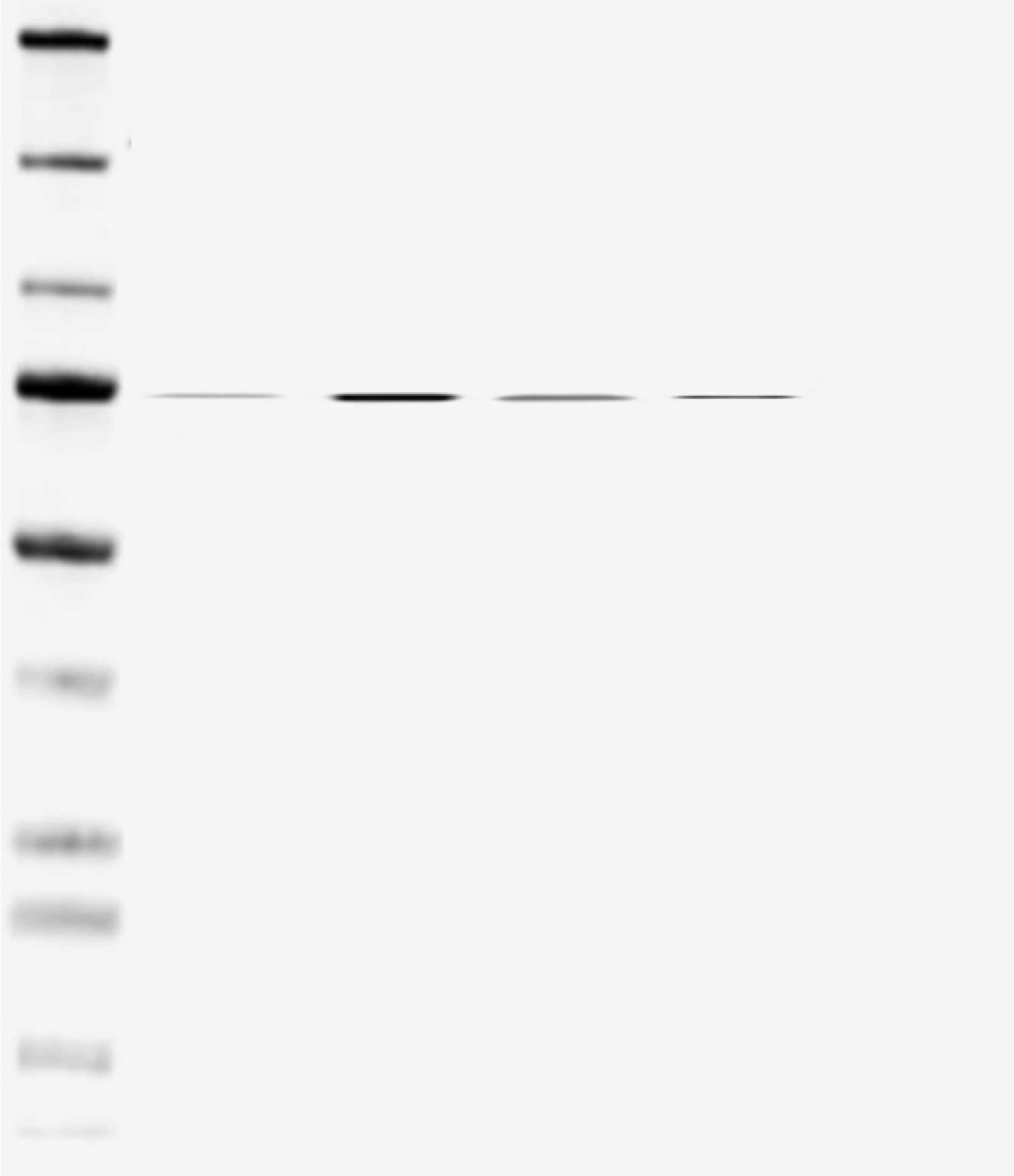

Brain NF-κB p50

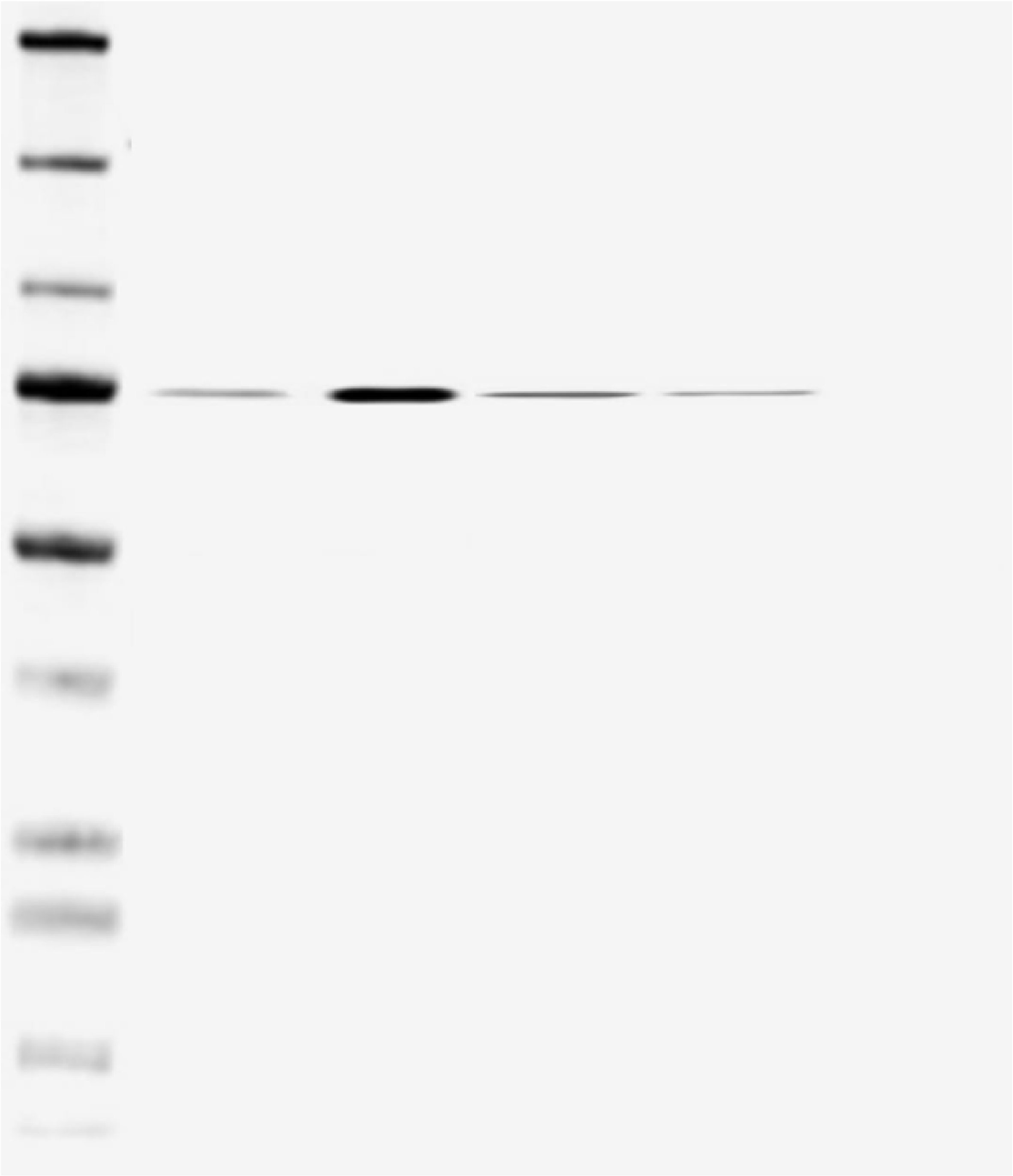

Brain NF-κB p50

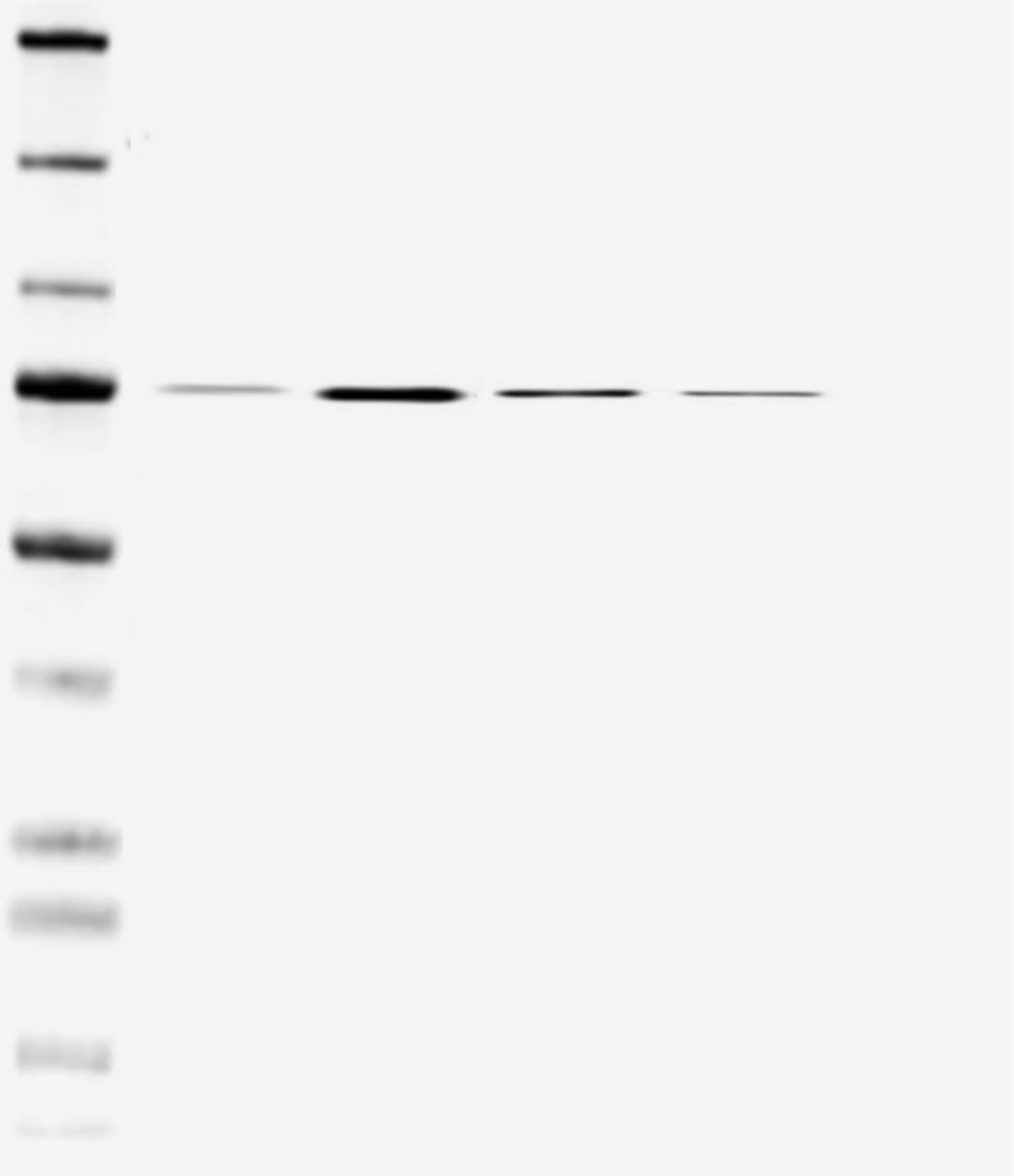

Supplement: Supplementary file 1 — (PDF 227 KB) [file 210_2026_5019_MOESM1_ESM.pdf]
