# Supplementary material for: Repurposing lurasidone to alleviate doxorubicin-induced cardiotoxicity and neurotoxicity via BDNF/TrkB/PI3K/Akt/CREB and miR-34a-5p/PGC-1α pathways
Source: Naunyn Schmiedebergs Arch Pharmacol. 2026 Mar 31;399(9):13731–57. doi: 10.1007/s00210-026-05019-z (PMC13357379; doi:10.1007/s00210-026-05019-z)

TR KB 145 KD a

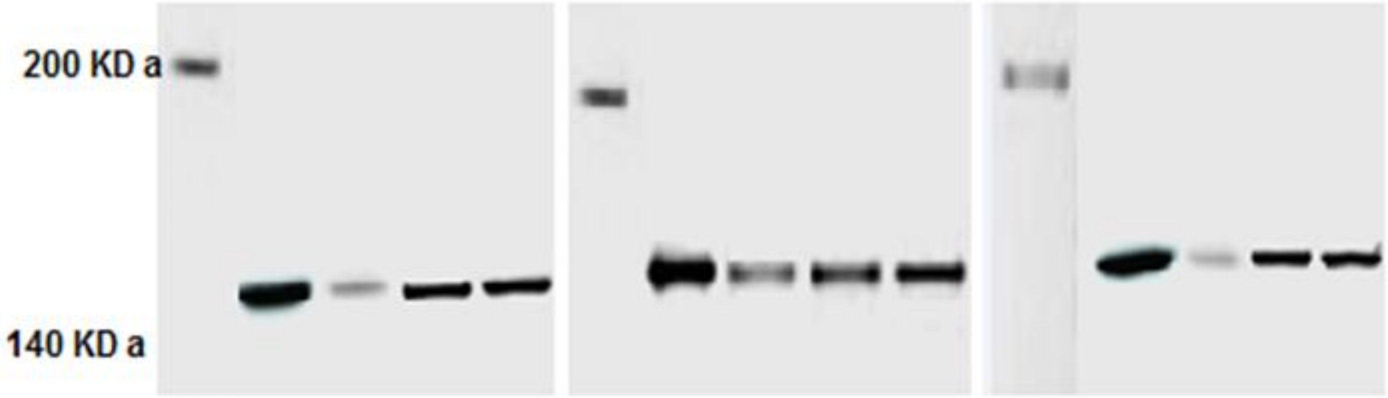

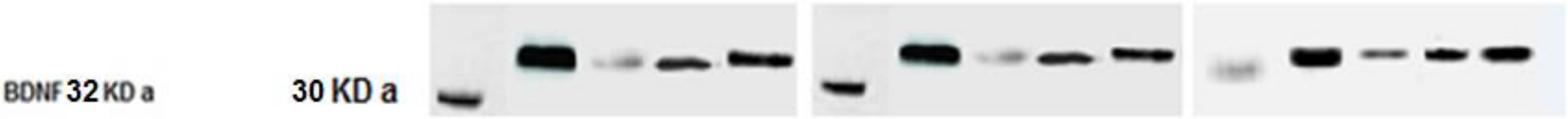
BDNF 32 KD a
30 KD a

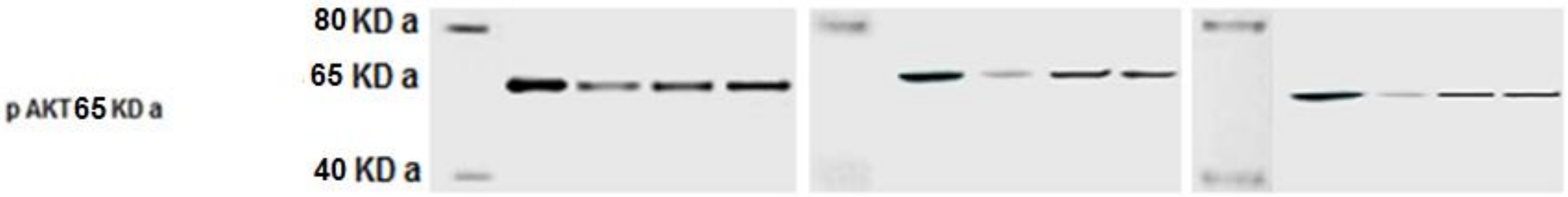
p AKT 65 KD a
80 KD a
65 KD a
40 KD a

p creb 43 KD a

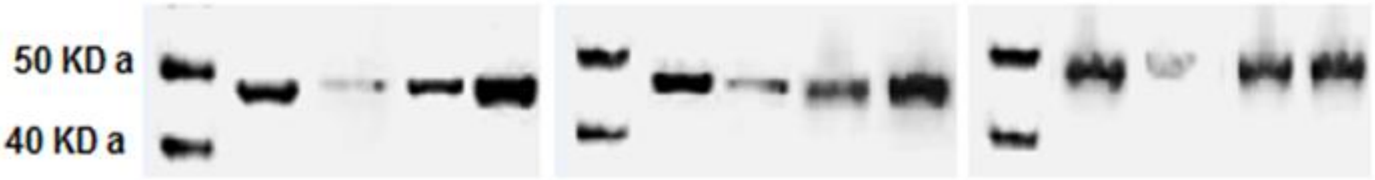

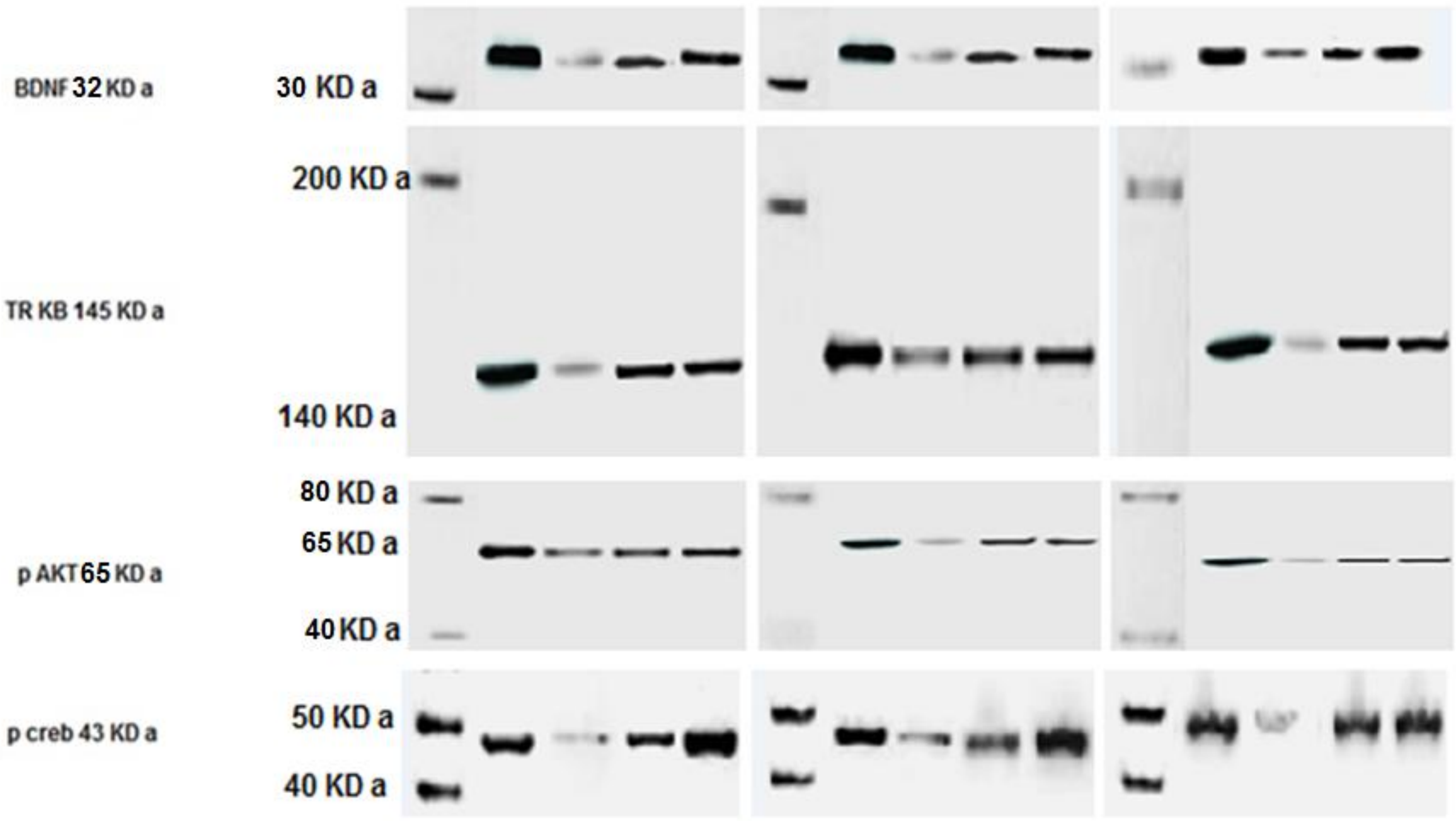
BDNF 32 KD a
30 KD a
200 KD a
TR KB 145 KD a
140 KD a
80 KD a
65 KD a
p AKT 65 KD a
40 KD a
50 KD a
p creb 43 KD a
40 KD a

β- actin heart

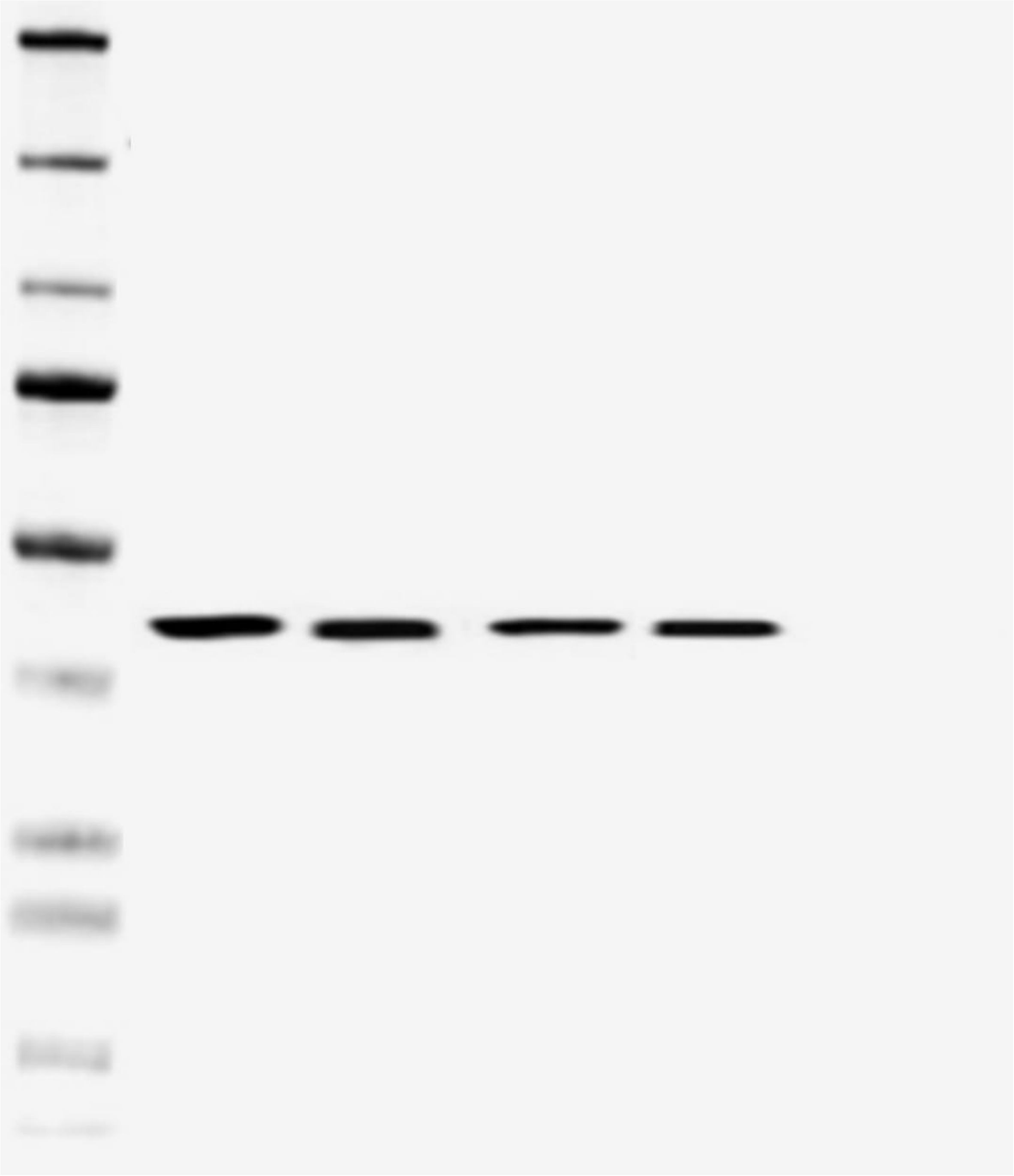

β- actin heart

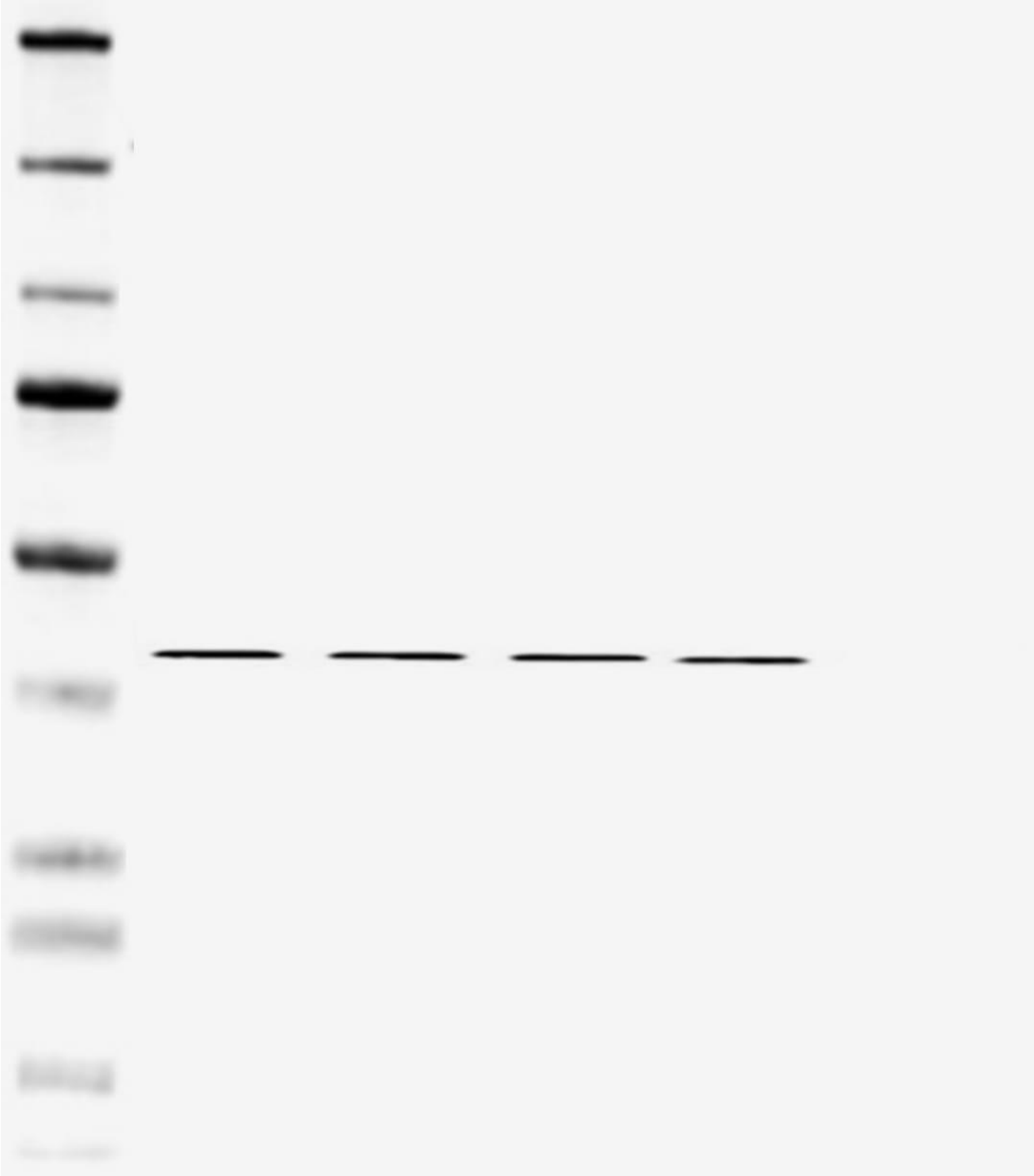

β- actin heart

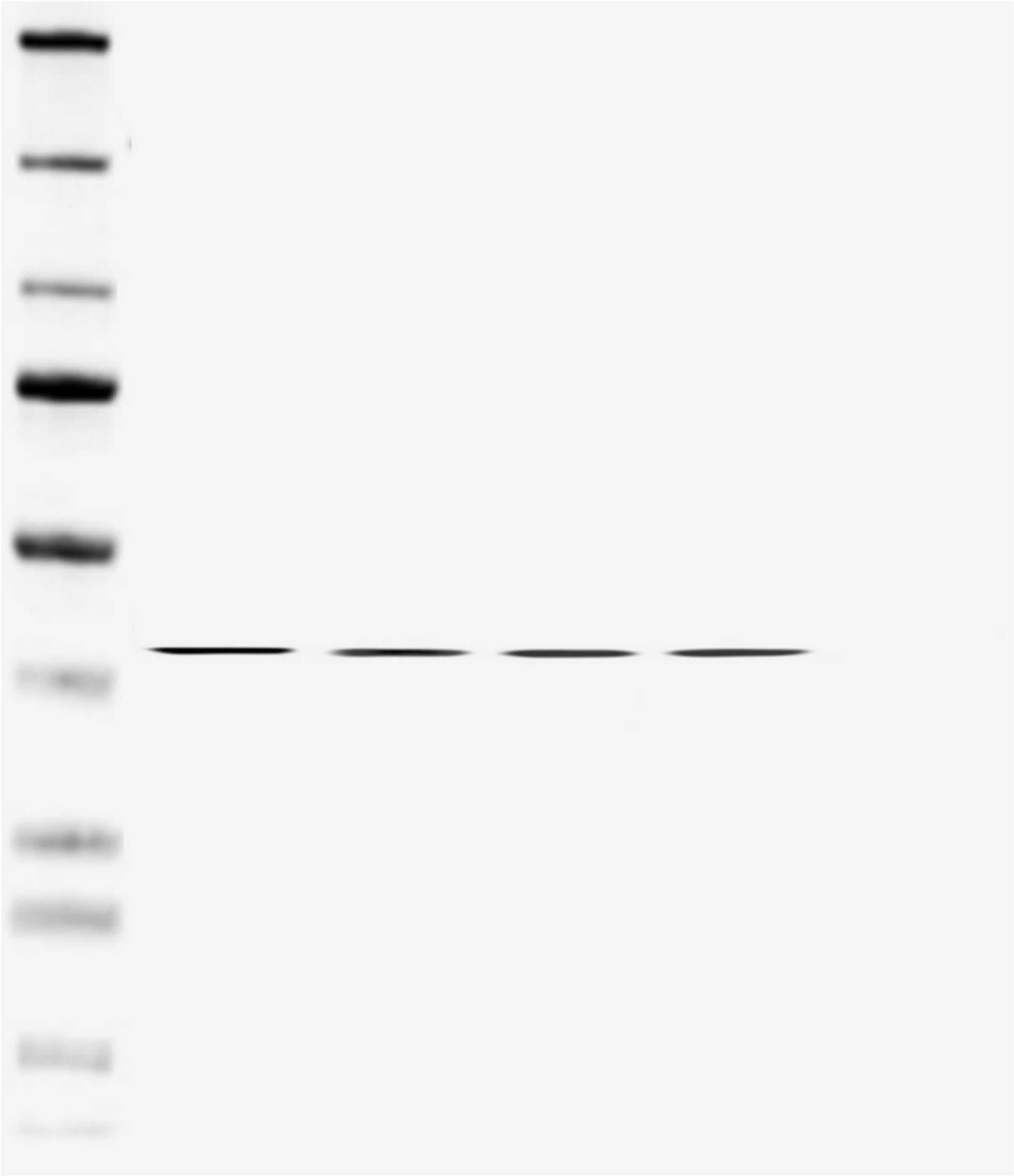

NF-KB p50
Heart

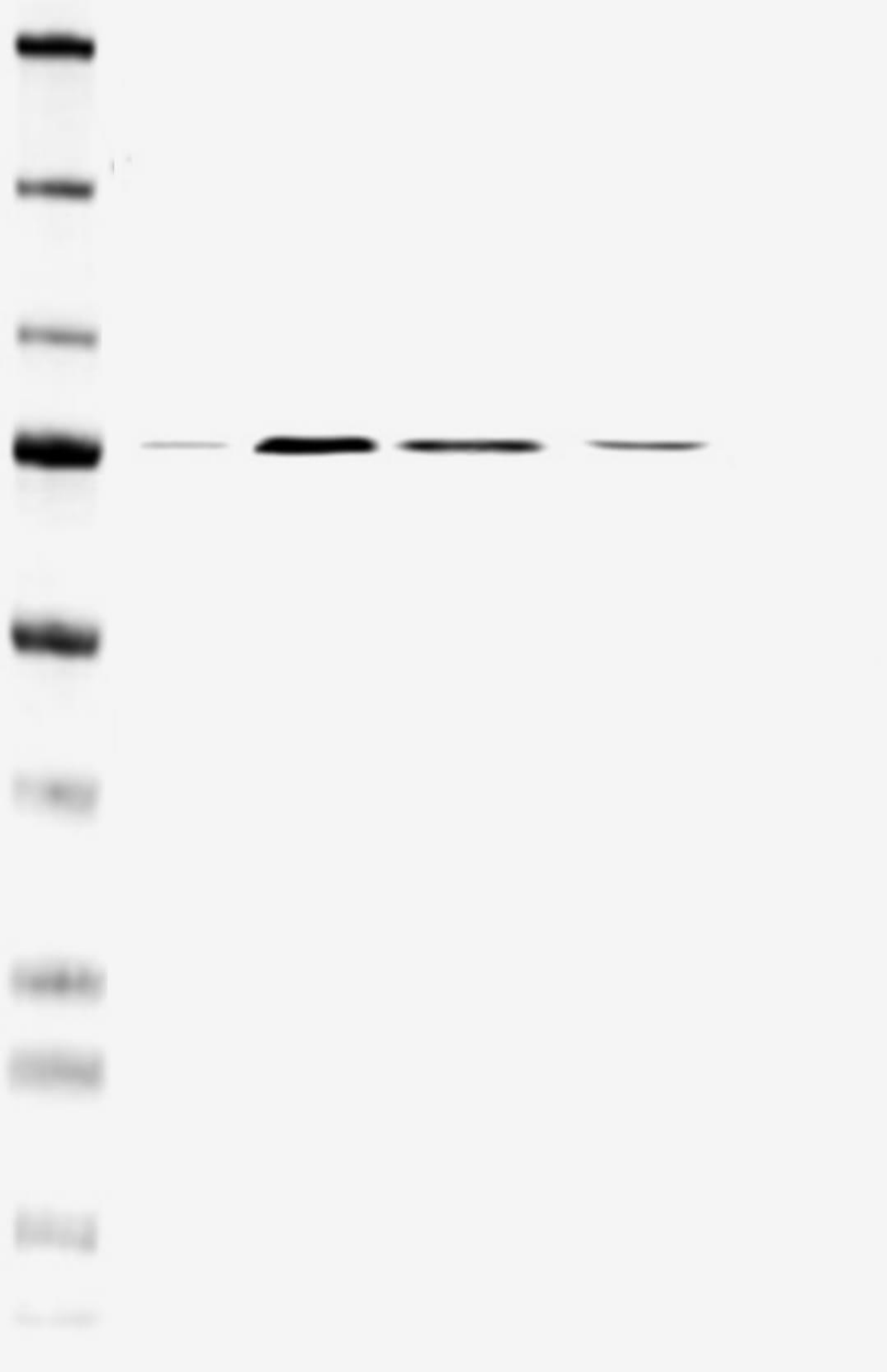

NF-KB p50
Heart

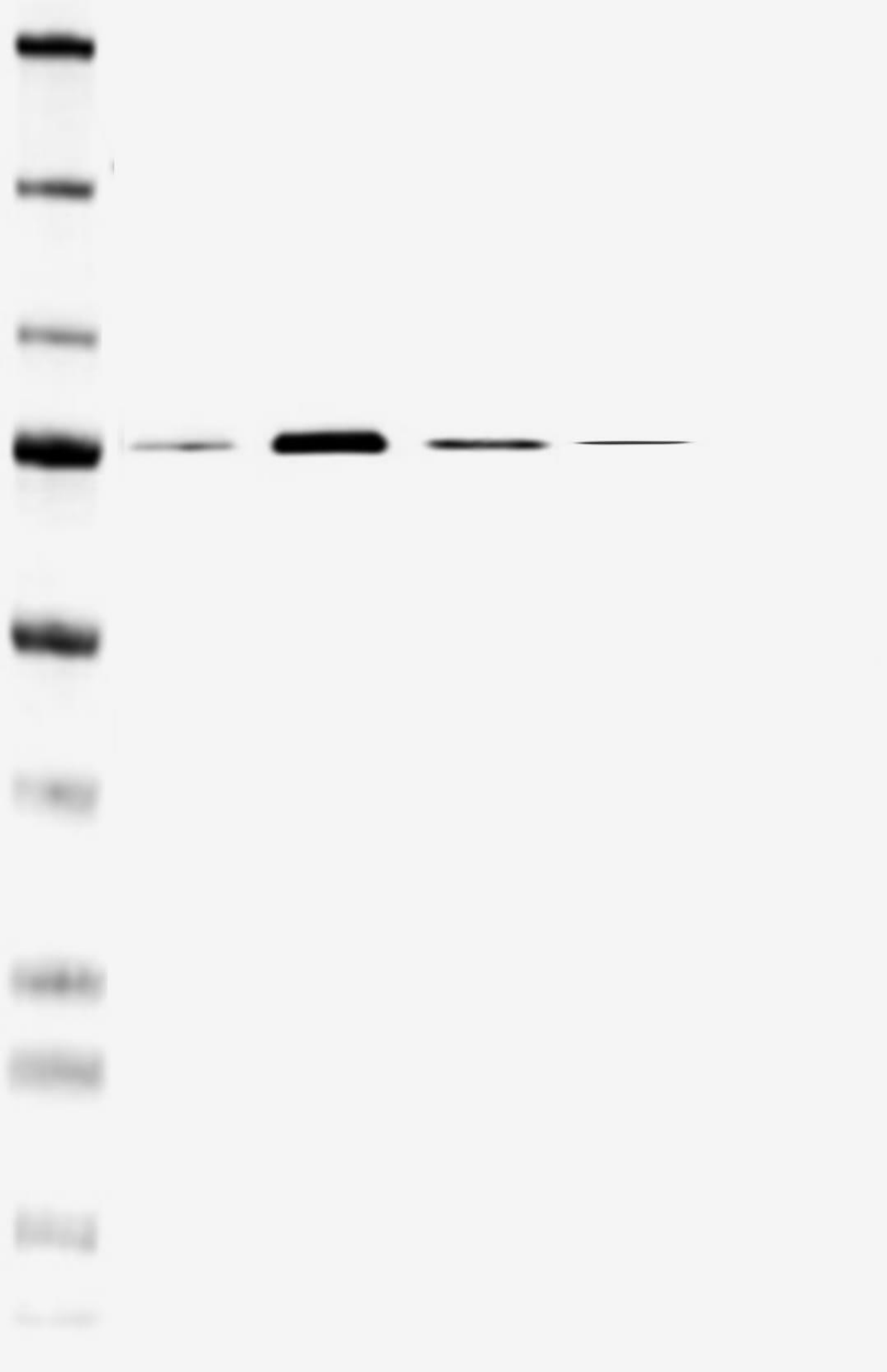

NF-KB p50
Heart

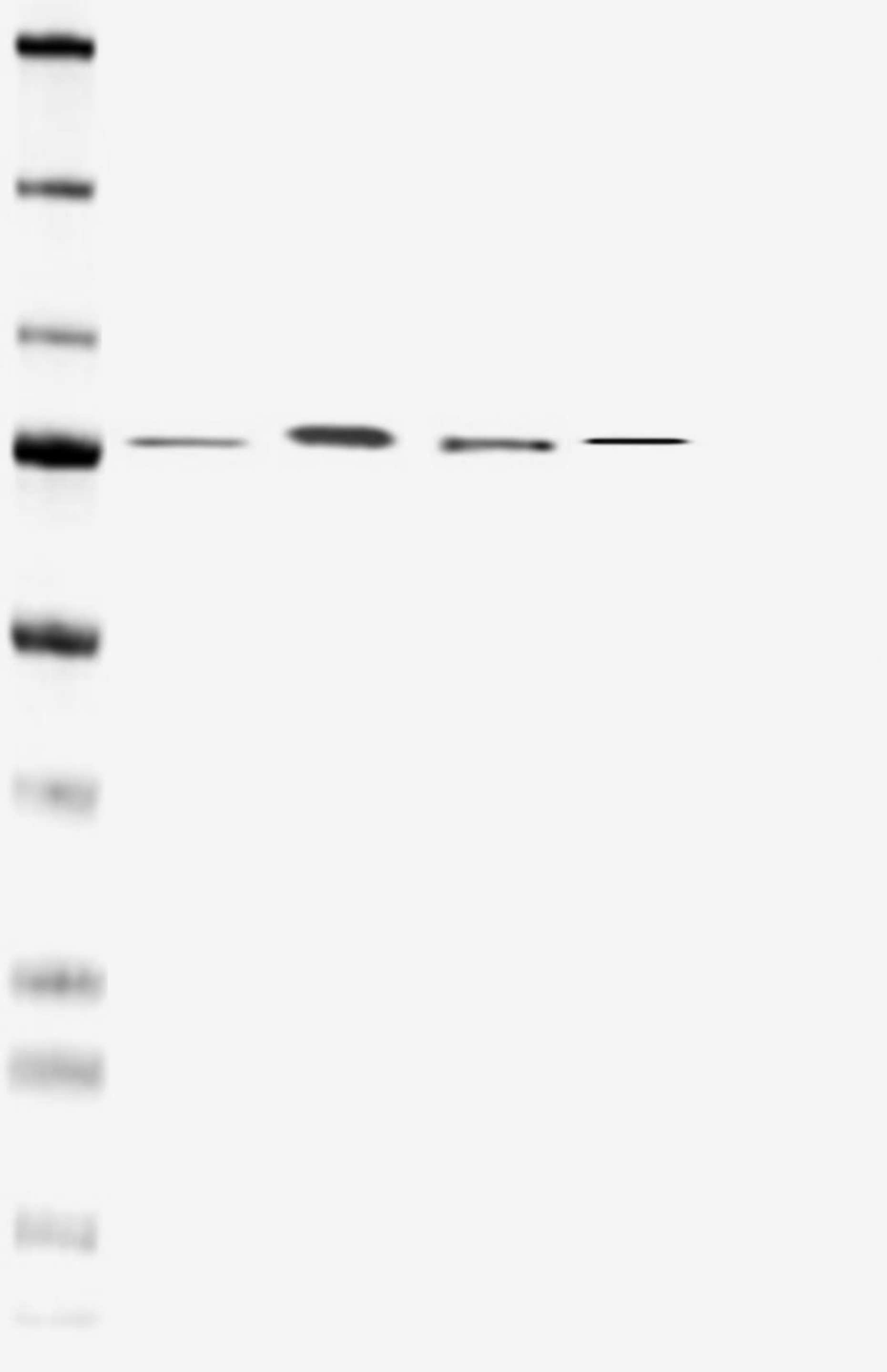

Supplement: Supplementary file 2 — (PDF 273 KB) [file 210_2026_5019_MOESM2_ESM.pdf]
